# Supplementary material for: Advanced machine learning approaches for predicting Neglected Tropical Disease co-endemicity in Kenya: A focus on soil-transmitted helminths, schistosomiasis, and lymphatic filariasis
Source: PLoS Negl Trop Dis. 2026 Mar 30;20(3):e0014156. doi: 10.1371/journal.pntd.0014156 (PMC13046276; doi:10.1371/journal.pntd.0014156)
Supplement: Attachment — Submitted filename: Review-D-25-01087.pdf [file pntd.0014156.s004.pdf]

PLOS Neglected Tropical Diseases

Advanced Machine Learning Approaches for Predicting Neglected Tropical Disease (NTD) Co-endemicity in Kenya: A Focus on Soil-Transmitted Helminths, Schistosomiasis, and Lymphatic Filariasis.

--Manuscript Draft--

**Manuscript Number:**	PNTD-D-25-01087R1
**Full Title:**	Advanced Machine Learning Approaches for Predicting Neglected Tropical Disease (NTD) Co-endemicity in Kenya: A Focus on Soil-Transmitted Helminths, Schistosomiasis, and Lymphatic Filariasis.
**Short Title:**	Advanced Machine Learning Approaches for Predicting NTDS Co-endemicity in Kenya
**Article Type:**	Research Article
**Keywords:**	Co - endemicity; neglected tropical diseases; XG Boost; random forest; Gradient Boosting Machine; wash; ESPEN
**Abstract:**	Background<br><br>Neglected Tropical Diseases (NTDs) affect 1.5 billion people worldwide with 39% of the global burden occurring in Africa. In Kenya, NTDs remain endemic despite control efforts, with co-endemicity of soil-transmitted helminths (STH), schistosomiasis (SCH), and lymphatic filariasis (LF) complicating intervention strategies. This study developed machine learning models to predict high-risk co-endemic areas using demographic and Water, Sanitation, and Hygiene (WASH) indicators.<br><br>Methodology<br><br>The study analyzed Kenya's 2022 NTD co-endemicity data from the Expanded Special Project for Elimination of Neglected Tropical Diseases (ESPEN), incorporating WASH and population variables. Three machine learning algorithms—Random Forest (RF), Gradient Boosting Machine (GBM), and XGBoost were trained to classify regions by STH prevalence levels and co-endemicity status. Model performance was evaluated using cross-validation, ROC-AUC, and variable importance analysis.<br><br>Results<br><br>The RF model achieved the highest predictive performance (AUC = 0.70), followed by XGBoost (AUC = 0.66) and GBM (AUC = 0.62). Key predictors included improved sanitation access (mean importance score: 0.24), population density (0.21), and co-endemicity with LF/SCH (0.18). Spatial analysis identified Eastern and North-Eastern Kenya as persistent hotspots, correlating with low WASH coverage (<40%).<br><br>Conclusion<br><br>Machine learning models effectively identified the high-risk NTD co-endemic areas in Kenya, with RF outperforming other models. These findings support targeted interventions integrating WASH improvements with mass drug administration (MDA) in identified hotspots. We propose a real-time dashboard for dynamic risk mapping to optimize resource allocation, a strategy aligned with Kenya's NTD Elimination Strategic Plan 2030.
**Additional Information:**	
**Question**	**Response**
**Government Employee**<br><br>Are you or any of the contributing authors an employee of the United States government?	No - No authors are employees of the U.S. government.

Powered by Editorial Manager® and ProduXion Manager® from Aries Systems Corporation

Manuscripts authored by one or more US Government employees are not copyrighted, but are licensed under a CC0 Public Domain Dedication, which allows unlimited distribution and reuse of the article for any lawful purpose. This is a legal requirement for US Government employees.<br><br>This will be typeset if the manuscript is accepted for publication.	
**Financial Disclosure**<br><br>Enter a financial disclosure statement that describes the sources of funding for the work included in this submission. Review the submission guidelines for detailed requirements. View published research articles from *PLOS Neglected Tropical Diseases* for specific examples.<br><br>This statement is required for submission and **will appear in the published article** if the submission is accepted. Please make sure it is accurate.<br><br>**Funded studies**<br>Enter a statement with the following details:<br>• Initials of the authors who received each award<br>• Grant numbers awarded to each author<br>• The full name of each funder<br>• URL of each funder website<br>• Did the sponsors or funders play any role in the study design, data collection and analysis, decision to publish, or preparation of the manuscript?<br><br>Did you receive funding for this work?	The author(s) received no specific funding for this work.
**Competing Interests**<br><br>On behalf of all authors, disclose any competing interests that could be perceived to bias this work.	The authors have declared that no competing interests exist.

Powered by Editorial Manager® and ProduXion Manager® from Aries Systems Corporation

This statement will be typeset if the manuscript is accepted for publication.<br><br>Review the instructions link below and PLOS NTDs' competing interests policy to determine what information must be disclosed at submission.	
**Data Availability**<br><br>Provide a **Data Availability Statement** in the box below. This statement should detail where the data used in this submission can be accessed. This statement will be typeset if the manuscript is accepted for publication.<br><br>Before publication, authors are required to make all data underlying their findings fully available, without restriction. Review our PLOS Data Policy page for detailed information on this policy. Instructions for writing your Data Availability statement can be accessed via the Instructions link below.	The data used in this study is publicly available via the link https://espen.afro.who.int/

Powered by Editorial Manager® and ProduXion Manager® from Aries Systems Corporation

Manuscript Click here to access/download;Manuscript;Manuscript.docx

Advanced Machine Learning Approaches for Predicting Neglected Tropical Disease (NTD) Co-endemicity in Kenya: A Focus on Soil-Transmitted Helminths, Schistosomiasis, and Lymphatic Filariasis.

Nkuba Nyerere [1], Damaris Felistus Mulwa[1, 2]

[1]Department of Mathematics and Statistics

Sokoine University of Agriculture, Morogoro, Tanzania

[2]Department of Statistics and Actuarial Sciences

Jomo Kenyatta University of Agriculture and Technology, Nairobi, Kenya

Corresponding author: Damaris Felistus Mulwa[1, 2]

Email: damaris.mulwa@jkuat.ac.ke

Abstract

Background: Neglected Tropical Diseases (NTDs) affect 1.5 billion people worldwide with 39% of the global burden occurring in Africa. In Kenya, NTDs remain endemic despite control efforts, with co-endemicity of soil-transmitted helminths (STH), schistosomiasis (SCH), and lymphatic filariasis (LF) complicating intervention strategies. This study developed machine learning models to predict high-risk co-endemic areas using demographic and Water, Sanitation, and Hygiene (WASH) indicators.

Methodology: The study analyzed Kenya's 2022 NTD co-endemicity data from the Expanded Special Project for Elimination of Neglected Tropical Diseases (ESPEN), incorporating WASH and population variables. Three machine learning algorithms, Random Forest (RF), Gradient Boosting Machine (GBM), and Extreme Gradient Boosting (XGBoost) were trained to classify regions by STH prevalence levels and co-endemicity status. Model performance was evaluated using cross-validation, Receiver Operating Characteristic (ROC) – Area under the Curve (AUC) and variable importance analysis.

Results: The RF model achieved the highest predictive performance (AUC = 0.70), followed by XGBoost (AUC = 0.66) and GBM (AUC = 0.62). Key predictors included improved sanitation access (mean importance score: 0.24), population density (0.21), and co-endemicity with LF/SCH (0.18). Spatial analysis identified Eastern and North-Eastern Kenya as persistent hotspots, correlating with low WASH coverage (<40%).

Conclusion: Machine learning models effectively identified the high-risk NTD co-endemic areas in Kenya, with RF outperforming other models. These findings support targeted interventions integrating WASH improvements with mass drug administration (MDA) in identified hotspots. We propose a real-time dashboard for dynamic risk mapping to optimize resource allocation, a strategy aligned with Kenya's NTD Elimination Strategic Plan 2030.

Keywords: Co - endemicity, Neglected Tropical Diseases, XG Boost, RF, Gradient Boosting Machine, WASH, ESPEN.

Author summary

NTDs are a diverse group of communicable diseases affecting 1.5 billion people globally. In Africa, where about 39% of the world's NTD burden occurs, this study effectively used machine learning (ML) models to identify, map and forecast high-risk areas for NTD co-endemicity. The study specifically examined the co-occurrence of lymphatic filariasis (LF), schistosomiasis (SCH), and soil-transmitted helminths (STH). The results show that machine learning models in particular, the random forest are useful instruments for dynamic risk mapping of regions where NTD co-endemicity is present. The findings highlight how important the Water, Sanitation, and Hygiene (WASH) indicators are in addition to conventional mass drug administration (MDA) whereas the spatial analysis identified Eastern and North-Eastern regions as the persistent hotspots, correlating with low WASH coverage (<40%). In accordance with Kenya's NTD Elimination Strategic Plan 2030, the study findings suggest putting in place a real-time dashboard for ongoing risk monitoring and surveillance in order to facilitate focused interventions and maximize resource allocation.

1. Introduction

Co - endemicity refers to the geographic overlap of two or more NTDs in the same population [1]. NTDs are a diverse group of communicable diseases affecting 1.5 billion people globally [2]. Furthermore, the African population's bear 39% of the total global burden of NTDs and, of this, 90% is accounted for by five diseases (LF, Onchocerciasis, STH, SCH and Trachoma) which are treated through regular preventive chemotherapy (PC-NTDs) in a mechanism called mass drug administration (MDA) [3]. Up to 44 countries in Africa are endemic for at least 1 PC-NTD, 42 for at least 2 PC-NTDs and 17 for all the 5 PC-NTDs [4]. These preventable and treatable diseases cause severe pain, stigma and disfigurement while killing over 170,000 people and costing developing economies billions of dollars every year [5]. In Africa, only eight countries have eliminated at least one PC-NTD [6]. Despite the intervention control strategies, Kenya remains endemic of LF, SCH and STH. These infections, especially the STH, remain endemic especially in the rural areas with inadequate water and sanitation supply [7]. Despite progress in mass drug administration (MDA) campaigns, persistent transmission hotspots necessitate refined surveillance and intervention strategies.

LF is commonly known as elephantiasis and is a painful and profoundly disfiguring disease [8]. In endemic countries, the disease has a major social and economic impact [9]. It is primarily caused by three species of parasitic roundworms; *Wuchereria bancrofti* (the most common, and only species present in Africa), *Brugia malayi*, and *Brugia timori* [10]. These parasites are transmitted to humans through the bites of infected mosquitoes. Once inside the human body, the larvae develop into adult worms in the lymphatic system [11]. The key statistics for 2022 LF transmissions reveal that the population living in the implementation units was 4,257,611 people [3]. The population living in the implementation units and had stopped preventive chemotherapy was 319,806 while the population targeted for the preventive chemotherapy was 3,721,891.

STH are caused by parasitic worms and are widespread in areas with inadequate sanitation [12]. They primarily affect the intestines, leading to a range of health issues, with the most severe impacts on children [13]. Control and prevention efforts focus on improving hygiene and sanitation, as well as regular deworming treatments [14]. The main worms causing soil-transmitted helminthiases are roundworm (*Ascaris lumbricoides*), whipworm (*Trichuris*

trichiura), and hookworms (*Necator americanus* and *Ancylostoma duodenale*) [15]. These parasites are prevalent in areas with poor sanitation and are primarily transmitted through soil contaminated with human feces. In Kenya, the key statistics for STH transmission in 2022 showed that the population living in the implementation units and requiring preventive chemotherapy was 1,884,837 pre-school children and 4,180,094 school age children. The targeted population for preventive chemotherapy was 590,377 pre-school children and 1,556,647 school age children [3]. The population requiring preventive chemotherapy had a geographic coverage of 27%.

SCH is also known as bilharzia and is caused by parasitic flatworms called schistosomes and is transmitted through contact with contaminated freshwater [16]. It can cause a wide range of chronic health problems, including damage to internal organs [16]. The main species causing schistosomiasis are *Schistosoma mansoni*, *Schistosoma haematobium*, and *Schistosoma japonicum* [17]. These species vary geographically and cause different forms of the disease. In Africa, the two most prevalent species are *S. mansoni* and *S. haematobium*. SCH is most prevalent in tropical and subtropical areas, especially in poor communities without access to safe drinking water and adequate sanitation [18]. It's estimated to affect millions of people worldwide. In 2022, the key statistics for SCH reveals that the population living in the implementation units and requiring preventive chemotherapy was 5,665,977 school age children and 3,351,000 adults. The total population targeted with preventive chemotherapy was 1,329,949 school age children and a geographic coverage of 32% required preventive chemotherapy [3].

The Kenya's Ministry of Health has implemented a Strategic Plan for control of NTDs through the National NTD control program [3]. STH transmission is strongly influenced by environmental and socioeconomic factors, including access to clean water, sanitation, and hygiene (WASH) practices [19]. It's co-endemicity with LF and SCH further exacerbates disease burden, as overlapping risk factors (e.g., poor sanitation, vector exposure) amplify transmission dynamics [20]. Current control efforts rely on geospatial mapping and periodic surveys, but these approaches may lack predictive precision for emerging high-risk zones. Machine learningoffers a promising tool for enhancing NTD surveillance by identifying nonlinear relationships between risk factors and disease prevalence [21]. Recent studies have applied ML to predict schistosomiasis in Côte d'Ivoire [22] and LF in India [23], but few have integrated co-endemicity and WASH data for co- endemicity risk modeling in Kenya.

Despite the ongoing efforts to control and eliminate NTDs, current surveillance and intervention strategies often rely on static maps, outdated prevalence data, and non-predictive models. This limits the timely identification of high-risk areas and fails to capture the dynamic co-endemic interactions between the common NTDs like STH, SCH, and LF, which frequently co-occur in the same vulnerable populations, particularly in Sub-Saharan Africa. While supervised machine learning methods and models have been increasingly applied in broader disease mapping and risk prediction, there is a significant gap in the integration of spatially explicit machine learning models to predict and classify the co-endemicity of multiple NTDs with demographic and WASH variables. There remains limited research on using machine learning techniques to model co-endemic transmission risks of STH, SCH, and LF in a unified framework that supports data-driven approaches while targeting interventions especially in Kenya. By leveraging Kenya's 2022 co-endemicity dataset from the ESPEN portal, this study provides actionable insights for optimizing NTDS co-endemicity and control programs.

The main objectives of this study were to develop machine learning models (RF, GBM, and XGBoost) to predict high STH prevalence and the co - endemicity status with LF and STH using demographic and WASH indicators, identify the key drivers of co-endemicity transmission and identify the high burden hotspots for targeted interventions.

2. Materials and Methods

2.1 Study area

The study focused on the co-endemicity status of LF, STH and SCH in Kenya in 2022. All the regions required preventive chemotherapy.

2.2 Population Description

In this study, secondary data analysis using the Expanded Special Project for Elimination of Neglected Tropical Diseases (ESPEN) in Kenya's co - endemicty data for the year 2022 was done. Kenya, on the east coast of Africa, is endemic for LF, Schistosomiasis and STH. The Ministry of Health implements a Strategic Plan for control of NTDs through the National NTD control program. This study analyzed data at both national and subnational levels (implementation units) including a total of 47 counties and 290 implementation units which was sufficient for modelling.

2.3 Ethics

The dataset used in this study is publicly available via the link https://espen.afro.who.int/; and does not require ethical approvals for the preparation of the manuscript. The ESPEN Portal enables health ministries and stakeholders to share, and exchange subnational program data, in support of the NTD control and elimination goals.

2.4 Data source and cleaning

The data used in this study is publicly available via the link https://espen.afro.who.int/; Electronic data capture, with standardized data collection forms, and automatic encrypted data upload to a secure central server. Form testing with specialists and stakeholders is responsible for conducting the surveys to ensure the form will collect quality data and adapted to the specificities of the diseases. Online dashboard setup for daily data visualization and near-real-time data cleaning using automated algorithms is available in the ESPEN portal. Each country own their data and have their own restrictions. In this study, 2022 Kenyan dataset was used.

Data cleaning was carried out in R software version 4.5.0. The dataset contained 37 variables which were exclusively selected using the variable importance criterion. The missing values for the diseases data were detected during cleaning and were replaced using the mean of the complete samples. Since the data was balanced (35:65), there was no need of re-sampling the minority class. The data was balanced to a moderate extent to perform the training process to build the co - endemicity prediction. Sections 2.5 and 2.6 give more details on the variables used in the study. Flowchart of the proposed models and methodology

The workflow for this research is illustrated in Figure 1 below where each process to be followed in outlined and linked to other subsequent processes. The workflow summarizes the data analysis processes the study will follow from the beginning to the end.

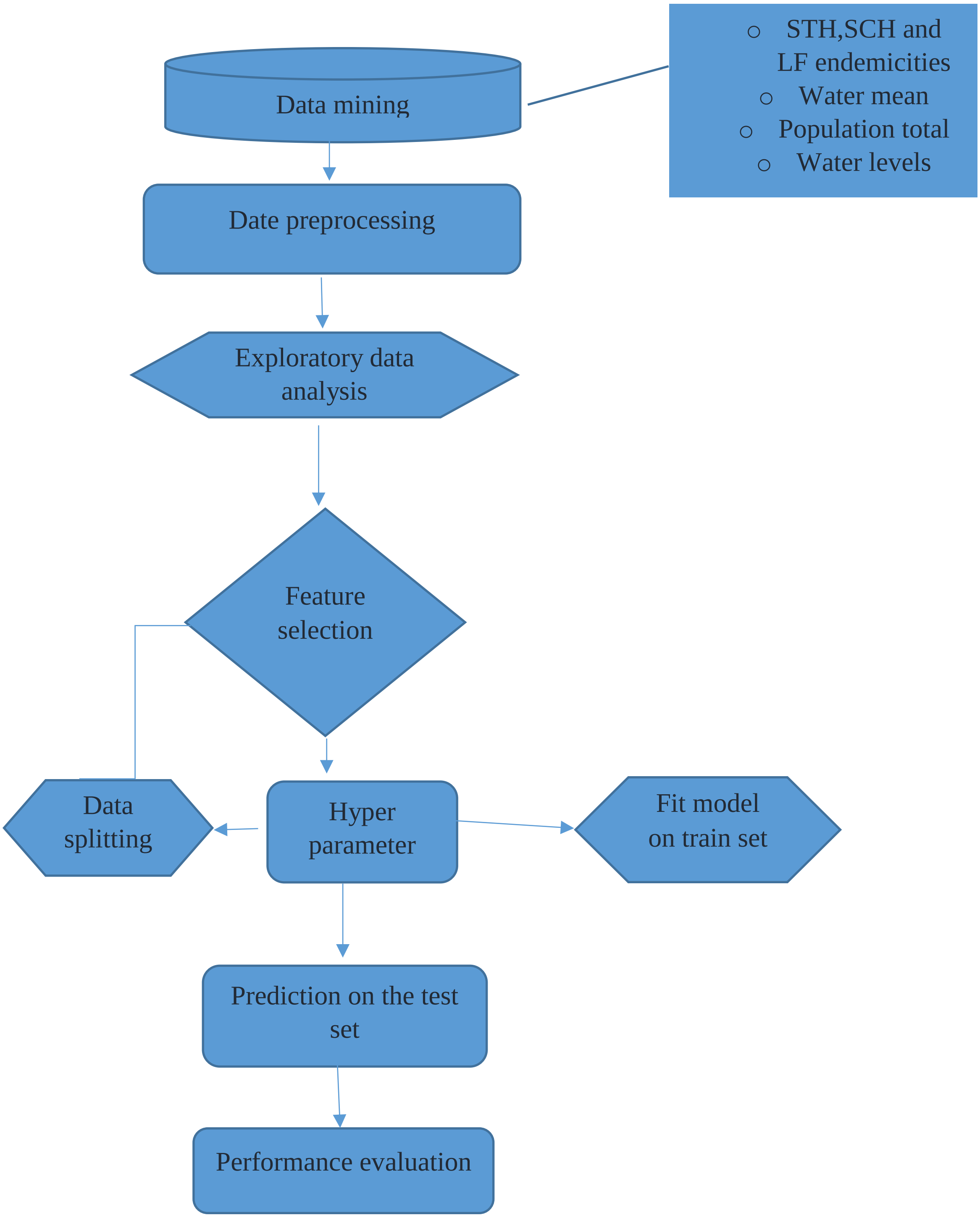

Figure 1: Flowchart for the proposed methodology

2.5 Outcome variable

The main target variable for this study was the co-endemicity status of the three NTDs (LF, STH and SCH) under consideration. Before the actual analysis using the machine learning algorithms, exploratory data analysis was conducted to understand the transmission

dynamics, patterns and the spatial distribution of the diseases. The raw data was transformed in order to be suitable for the machine learning methodology. Table 1 shows the key steps followed were the creation target variable, (co-endemicity class) whereby the disease endemicity status were combined into one categorical variable. One hot encoding was employed to convert categorical variables into binary outcomes for the machine learning compatibility.

Table 1: Creation of target variable. As stepwise procedure of how the target variable was created.

LF + STH +SCH	Co - endemic status of all the diseases
LF + STH	Co - endemic for LF and STH only
LF + SCH	Co - endemic for LF and SCH only
STH + SCH	Co - endemic for STH and SCH only
LF only, STH only, SCH only	Endemic for only one disease
Non endemic	No disease endemicity

2.6 Predictor Variables

The predictor variables used in the study were high STH (categorical variable), population total (continuous variable), improved water mean (continuous variable), improved sanitation mean (continuous variable), LF, SCH and STH endemicities (categorical variable), water levels (continuous variable) and the endemicity IDs (continuous variable). Comprehensive analysis of all the predictor variables is shown in the results section (3.1).

2.7 Statistical analysis

Statistical analysis was done using the latest version of R (4.5.0). The relevant R libraries (XGBoost, RF, pROC, GBM) were installed prior to the analysis. Correlation and spatial analysis were conducted to give more insights into the data. Data splitting was done in the ratio 80:20 for the training and testing roles. Data splitting is important in machine learning classifications and methodologies as it ensures model evaluation on unseen data, data structuring and handling, and avoids leakage before preprocessing. Model performance metrics and confusion matrix were used to evaluate the models. Confusion matrix shows the true versus the predicted classes. To identify the most significant predictors, variable importance analysis was fitted for each of the machine learning models.

2.3 Machine learning methodology

2.3.1 XGBoost Algorithm

The XGBoost model was used for enhanced prediction. The data was transformed to enhance the model fitting. The learning rate was 0.1 and the max_depth (tree depth) was set at 6. Early stopping was considered to prevent the model from over fitting and halting the training process in case the validation performance levels up. Feature importance by the XGBoost measures how each of the selected features contributes to the prediction process. The XGBoost algorithm handles the imbalanced data quite well as well as the large dataset.

2.3.2 RF Algorithm

RF is a supervised ML algorithm that builds multiple decision trees and merges their predictions to improve accuracy and control over fitting. In our study, the RF algorithm was used for both prediction and classification tasks. It combines predictions through classification (majority vote (for your High/Low endemicity classes) and regression (average prediction). ROC was used to select the optimal model using the largest value. The final value used for the model was mtry = 2.

2.3.3 Gradient Boosting Machine (GBM)

GBM is a sequential ensemble method that builds decision trees one at a time (unlike RF's parallel approach). Each new tree corrects errors of the previous ensemble and optimizes a loss function (e.g., deviance for classification) using gradient descent. The common tuning hyper parameters used in the current study includes an interaction. Depth = c (3, 5), n.trees = c (100, 200), shrinkage = c (0.01, 0.1) and n.minobsinnode = 10. Smaller shrinkage (≤ 0.1) with more n.trees (≥500) often performs better.

2.3.4 Ensemble Modelling

Ensemble modelling involves combinations of different predictions to improve the robustness. Predictions from the RF model, XGBoost and GBM are combined to yield a more robust model. The final prediction is termed as the one which is most frequent across the models. Ensemble modelling reduces bias and yields more stable predictions. Moreover, it outperforms the individual models.

2.8 Algorithm selection justification

The study chose only three machine learning algorithms namely RF, GBM and XGBoost. Firstly, the three algorithms are tree-based ensemble methods known for robustness to non-linear and complex relationships which are common in disease data, handling mixed data types i.e., categorical, continuous, discrete and without extensive feature scaling and lastly providing feature importance Scores (key for interpretability in disease modelling context). The Random forest and XGBoost are the leading tree-based ensembles selected for their well-documented performance on structured data and their ability to capture non-linearities.

3. Results

3.1 Exploratory data analysis

Figure 2 below shows the distribtion of the population at risk of the common NTDs in Kenya. The Y axis shows the regions while the X axis represents the population totals. It gives insights on the distribution and geographical loaction of the diseases under study. The Rift valley region had the highest population which is at risk of contacting the diseases. Nairobi and North eastern have the lowest population. Nairobi, being the capital city might have improved water and access to better sanitation services. North Eastern regions is relatively dry with little or no rainfall [24], hence the risk of disease transmission is low.

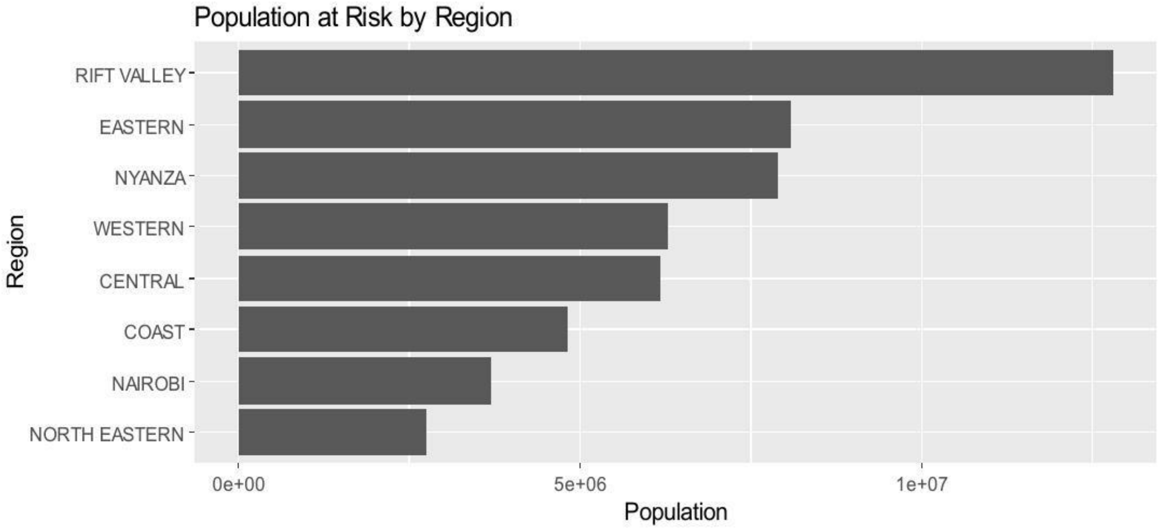

Figure 2: The population at risk of NTDs by region

Figure 3 shows the STH endemicity levels in Kenya. The STH endmicity level is categorized into high prevalence, low prevalence, moderate prevalence and non endemic. The low and moderate prevalences are the commonest in STH transmission. The Y axis shows the number of geographical units that falls under each category.

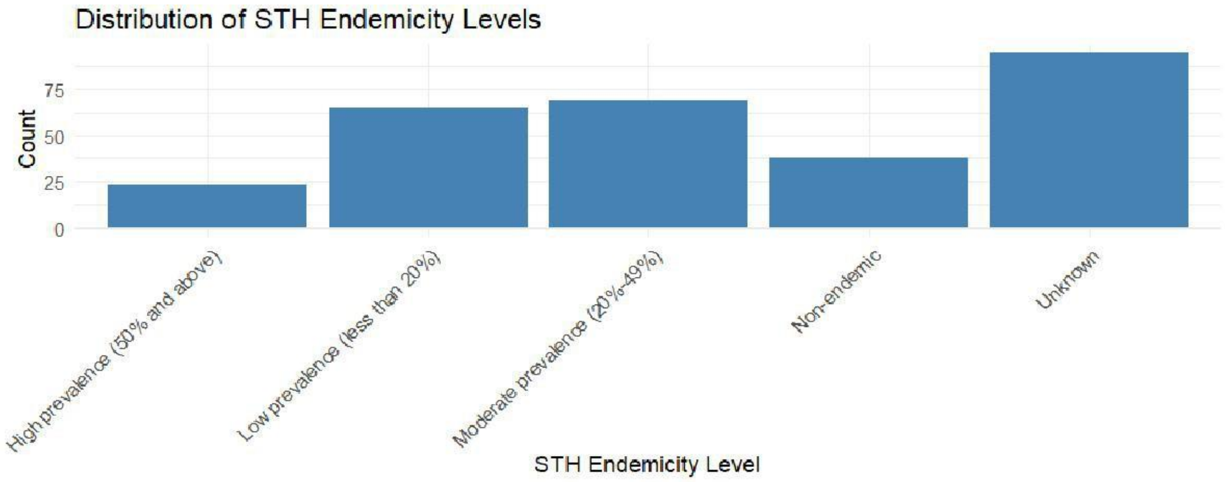

Figure 3: Distribution of STH endemicity levels

Figures 4 and 5 shows the distribution of SCH and LF endemicity levels respectively. LF is sparsely distributed while the SCH distribution is high in all the four categories (High, low, moderate and non-endemic). The prevalence levels are categorized as high prevalence (>50%), moderate prevalence (20-49%), low prevalence (<20%) and non-endemic (areas where transmission did not occur).

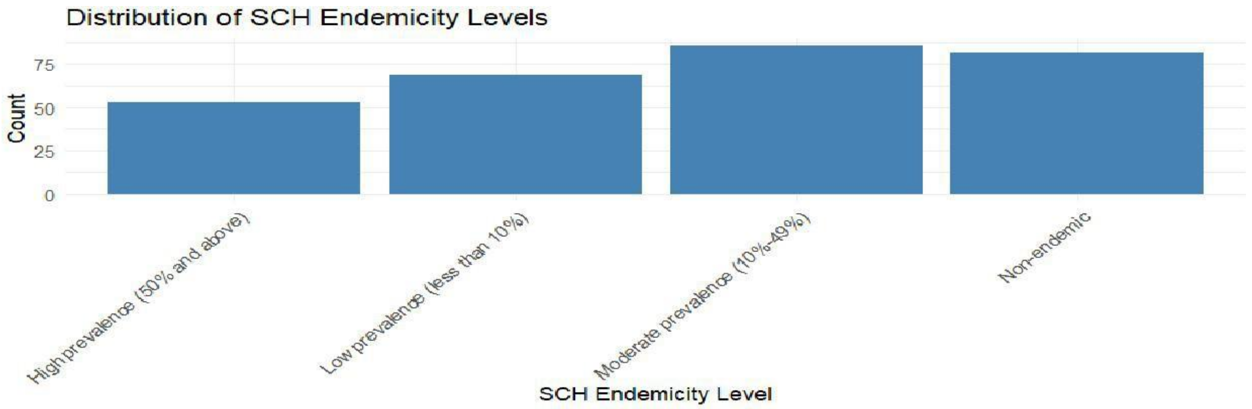

Figure 4: Distribution of SCH endemicity levels

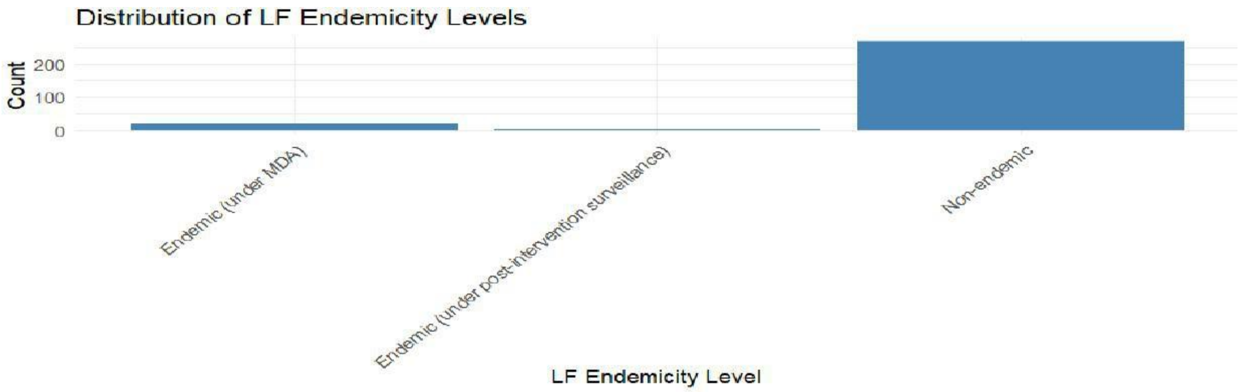

Figure 5: Distribution of LF endemicity levels

Figure 6 shows the co-endemicity patterns by region. The coastal region has high LF and STH co-endemicity while western region has high SCH and STH overlap. The North Eastern region has few disease hotspots. The effectiveness of the MDA by region is shown in Figure 7. T2 is the commonest in majority of the regions. T1 works best in high co-endemicity regions.

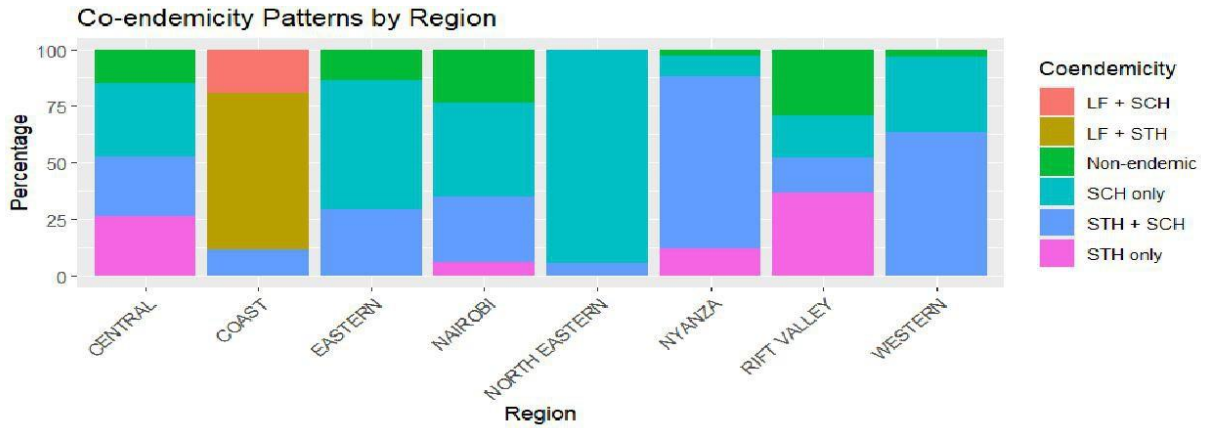

Figure 6: NTDs co-endemicity status by region

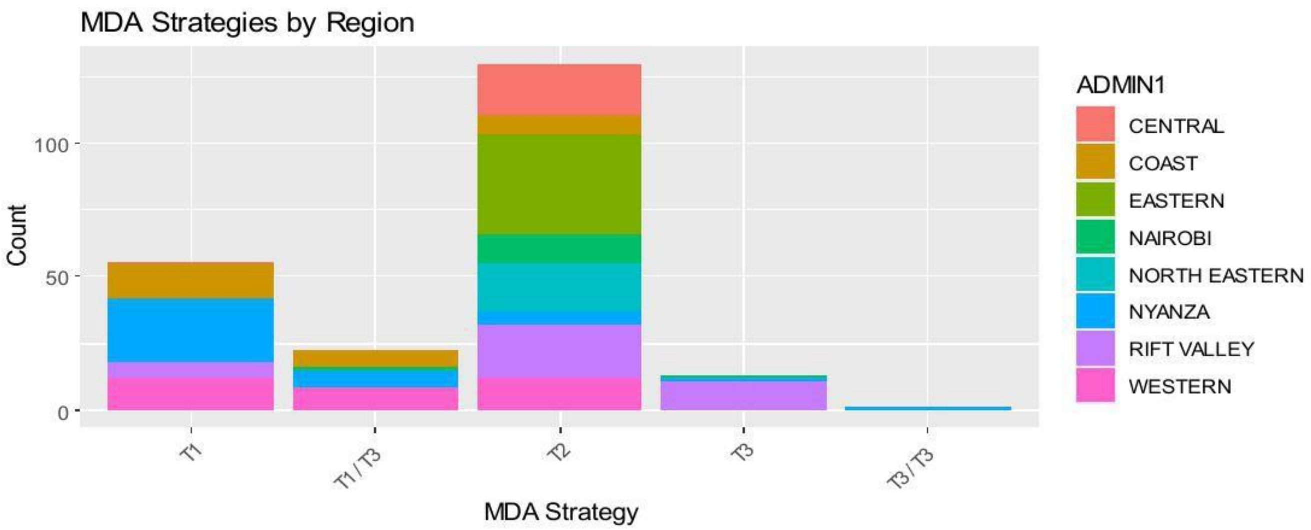

Figure 7: MDA strategies and usage by region

3.2 Correlation between the variables

Table 2 shows the correlation analysis of the relationships between the predictor variables (water and sanitation means, endemicity levels and the population total). Pearson's (r) and the spearman's (ρ) were used since the data was a mixture of both continuous and categorical variables.

The correlation matrix in Table 2 unveils some important relationships between the disease endemicity status, population structure and the WASH variables. The STH has a weak negative correlation with the population of the region and a very weak negative correlation with the water access. Moreover, the STH is positively correlated with the improved water sanitation. LF endemicity is positively correlated with water access but negatively correlated with sanitation. The SCH endemicity has a weak positive correlation with the population and positively correlated with all the other variables. The WASH variables, (water and sanitation) are positively correlated with other. Access to improved water shows a positive correlation with the diseases as compared to sanitation.

Table 2: Correlation matrix. The correlation matrix shows the relationship between the target variable and the predictors.

Variable	Pop Tot	STH Endemicity	Water mean	Sanitation mean	LF Endemicity	SCH Endemicity
**Pot Tot**	1.0000	-0.0183	0.3344	0.1752	0.0042	0.0334
**STH Endemicity**	-0.0183	1.0000	-0.0282	0.1234	-0.0590	0.0756
**Water mean**	0.3344	-0.0282	1.0000	0.3526	0.1623	0.1612

**Sanitation mean**	0.1752	0.1234	0.3526	1.0000	-0.1668	0.1301
**LF Endemicity**	0.0042	-0.0590	0.1623	-0.1668	1.0000	0.1607
**SCH Endemicity**	0.0334	0.0756	0.1612	0.1301	0.1607	1.0000

3.3 Spatial analysis of STH prevalence

Figure 8 shows the spatial analysis of STH prevalence across the regions. Eastern and North eastern regions have the highest STH endemicity while Rift valley, Nyanza, coast and Nairobi regions have the lowest. These regions are highly favored and have better access to sanitation, environmental exposure and improved health programs.

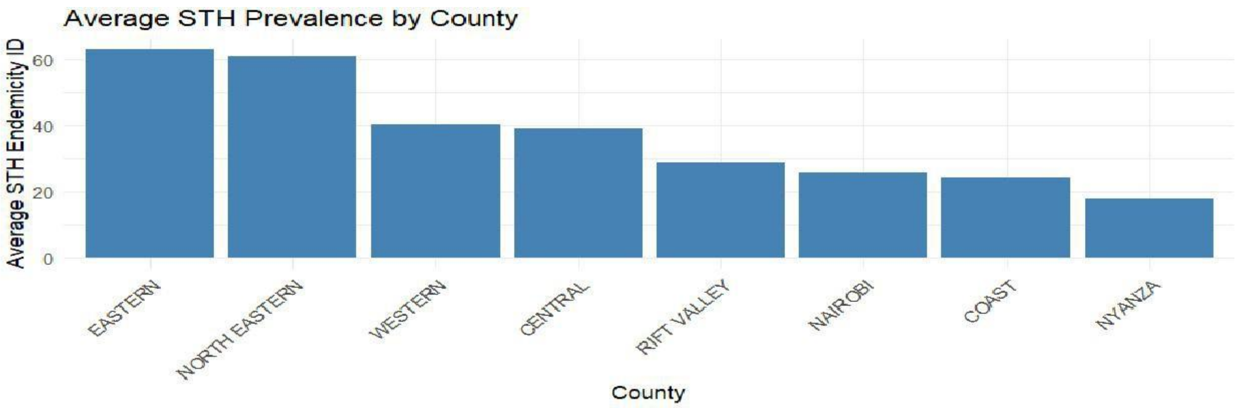

Figure 8: STH prevalence by region in Kenya

3.4 The RF model

The RF model was trained to classify areas as "High" or "Low" disease risk based on 7 predictors using 233 samples. The model's classification results are as shown in Table 3, Table 4, Figures 8, 9 and 10. ROC was used to select the optimal model using the largest value and the final value used for the model mtry (number of variables randomly sampled at each split) was 2. The class resampling cross validation was set at 5-fold.

Table 3 indicates that in the overall model, the predictive power was low since the ROC (0.5979) < 0.7. The sensitivity (0.6600) clearly indicates that only 66% of high-risk areas will be correctly identified and 53% (specificity) of low-risk areas will be misclassified as high-risk.

Table 3: Model overall performance metrics and statistics of the RF model

Mtry	ROC	Sensitivity	Specificity
**2**	0.5979	0.6600	0.4688

**3**	0.5851	0.6043	0.5346
**4**	0.5728	0.6040	0.5061
**5**	0.5511	0.5800	0.5061
**7**	0.5401	0.5800	0.4589

Table 4: RF model key performance metrics

**Metric**	**Value**
Sensitivity	0.8387
Accuracy	0.6842
Specificity	0.5000
Positive predicted	0.6667
Negative Predicted	0.7222
Prevalence	0.5439
Detection rate	0.4561
Detection prevalence	0.6842
Balanced accuracy	0.6694
Kappa	0.3473
No information rate (NIR)	0.5439
P value	0.0219
95% CI	0.5476, 0.8009

The RF model was plotted and shows the cross validation versus the number of predictors used as shown in Figure 9. The performance peaks at (0.59 – 0.60) predictive power and decreases across the predictors.

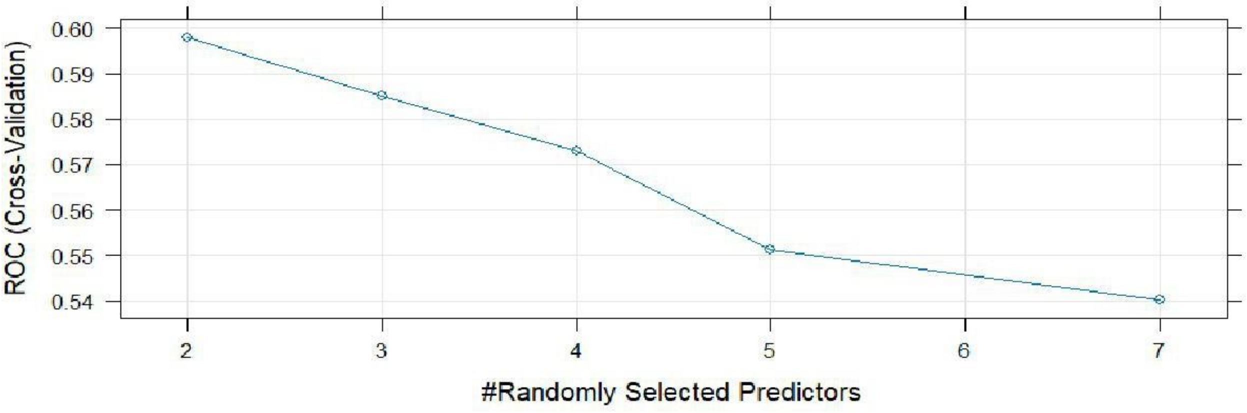

Figure 9: Random Forest plot

3.4.1 Variable importance features of the RF model

In Figure 10, the variable importance graph shows that the population of the region was the most important predictor while the water level (high low) was the least.

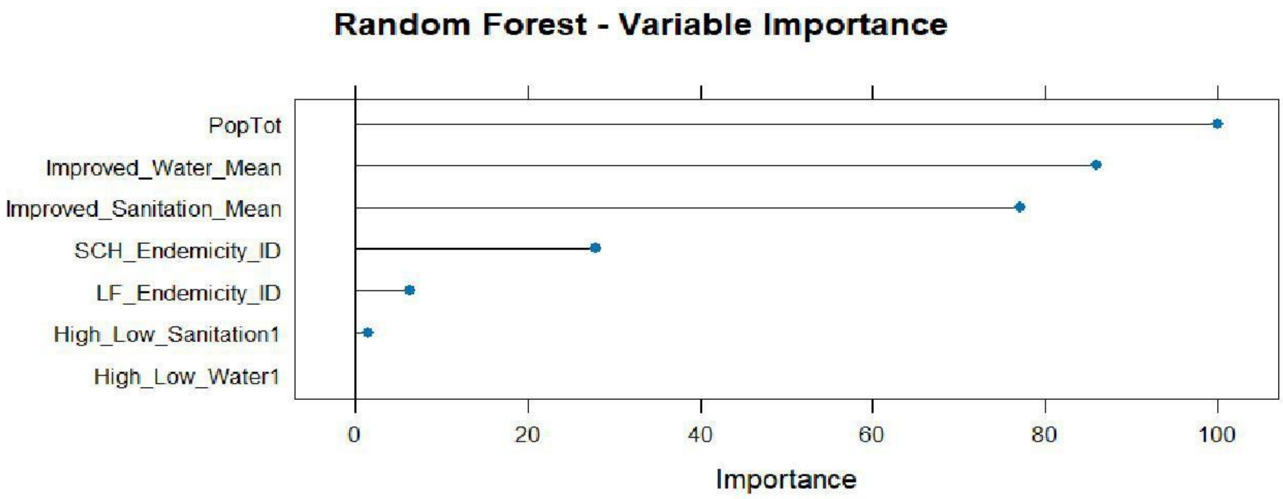

Figure 10: RF Variable importance

3.4.2 Confusion Matrix performance

A confusion matrix is a table used to evaluate the performance of a classification machine learning model. It shows how well your model's predicted classifications match the actual labels. The resulting confusion matrix for the RF model is shown in equation (1).

$$\begin{pmatrix} 26(TP) & 13(FP) \\ 5(FN) & 13(TN) \end{pmatrix} \quad \text{...} \quad (1)$$

The model correctly predicted 26 (TP) and 13 (TN) accounting for 68.42% (positive predicted) accuracy where TP (True positives), FP (False positive), FN (False Negative) and TN (True Negatives).

Table 4 shows that in the prediction task, the RF model accurately predicted 68.42% of the di seases cases. The wide confidence interval suggests a good variability in the model. The P - v alue (0.0219) was statistically significant and was better than random guessing (NIR = 54.39 %). The model's sensitivity (83.87%) means that it was able to detect the actual cases and wa s able to minimize false negatives. Only 50% of the actual low cases were correctly identified (specificity). The mean of sensitivity and specificity (balanced accuracy was 0.6694). The K appa, which is interpreted as a fair agreement between the variables was 0.3473.

Figure 11 shows the ROC curve of the RF model and it indicates that it had moderate predicti ve power (AUC = 0.703). A ROC curve is a graphical representation of a classification model 's performance at all possible classification thresholds. It helps you evaluate the trade-off bet ween sensitivity and specificity.

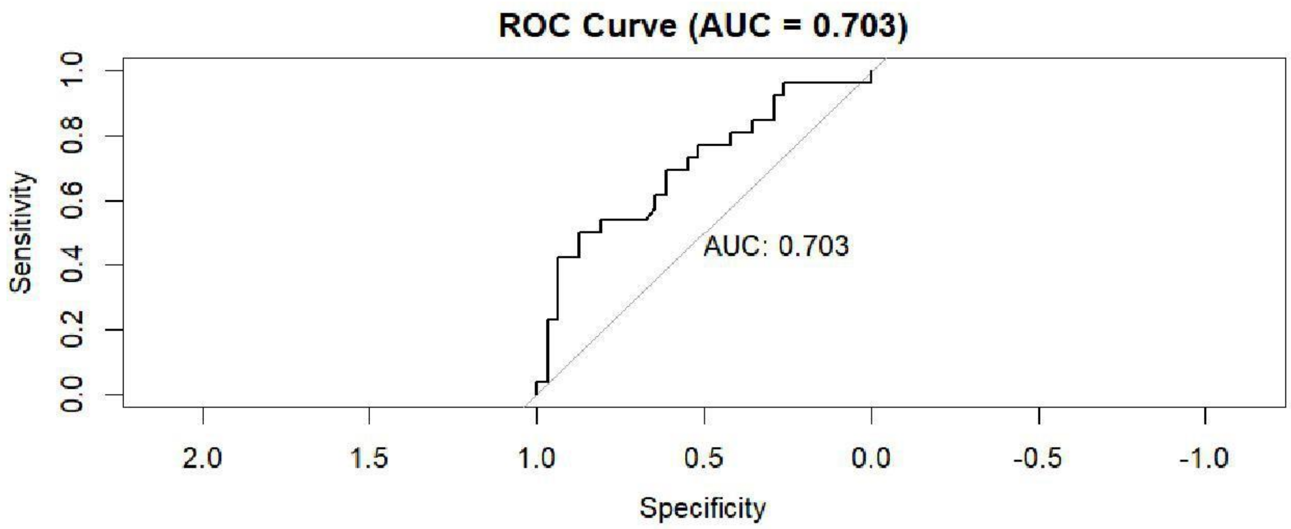

Figure 11: RF ROC AUC

Figure 11 shows the receiver operating characteristic, area under the curve (usually a plot of sensitivity against specificity) for the RF model.

3.5 The GBM model

GBM is a powerful ensemble learning algorithm model known for its strong predictive performance, especially in tabular data [25]. The resampling was set at 5 – fold cross validation with 7 predictors, 233 samples and 2 binary classes. The optimal hyper parameters were; n.trees = 50, interaction.depth = 2, shrinkage = 0.1, and n.minobsinnode = 10. The tuning parameter 'shrinkage' was held constant at a value of 0.1. Tuning parameter 'n.minobsinnode' was held constant at a value of 10. The ROC was used to select the optimal model using the largest value. The GBM model results are as shown in Figures 12, 13, 14 and Tables 5 and 6.
Figure 12 shows the results from the GBM hyper parameters. There is a competing performance from depths 1 to 3 with no clear winning case. There is an observable underperformance in the current model as compared to the RF.

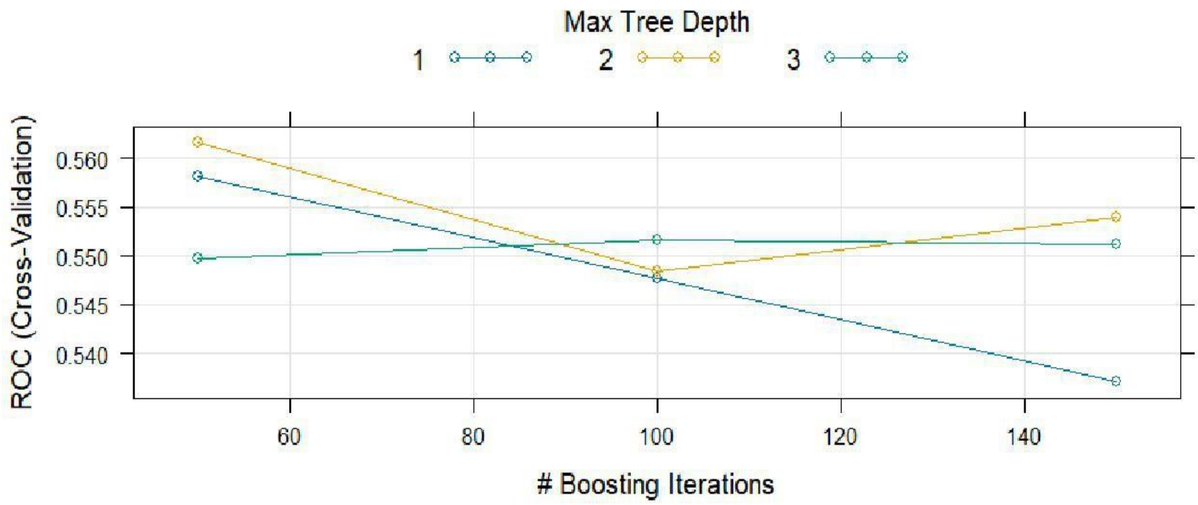

Figure 12: GBM model hyper parameter results

Figure 12 shows the hyper parameter results of the GBM model comprising of the maximum tree depths of different boosting iterations.

Table 5 shows the results of resampling across the tuning parameters. The best model was identified using the ROC metric (0.5691) and had $n.trees = 150$ and a depth of 2. The highest sensitivity (0.7068) was observed at interaction depth 1 and had a specificity of 0.3065. The final values used for the model were n.trees = 50, interaction.depth = 2, shrinkage = 0.1 and n.minobsinnode = 10.

Table 5: Resampling results across tuning parameters for the RF model.

Interaction depth	n.trees	ROC	Sensitivity	Specificity
1	50	0.4922	0.7068	0.3065
1	100	0.5444	0.6745	0.3723
1	150	0.5526	0.6748	0.4286
2	50	0.5230	0.6111	0.4273
2	100	0.5603	0.6108	0.4931
2	150	0.5691	0.6108	0.4641
3	50	0.5455	0.6114	0.3987
3	100	0.5598	0.6108	0.4732
3	150	0.5540	0.5945	0.5208

3.5.1 GBM variable importance

Figure 13 shows the variable importance of the GBM model. The total population, improved water and sanitation were identified as the most important predictors.

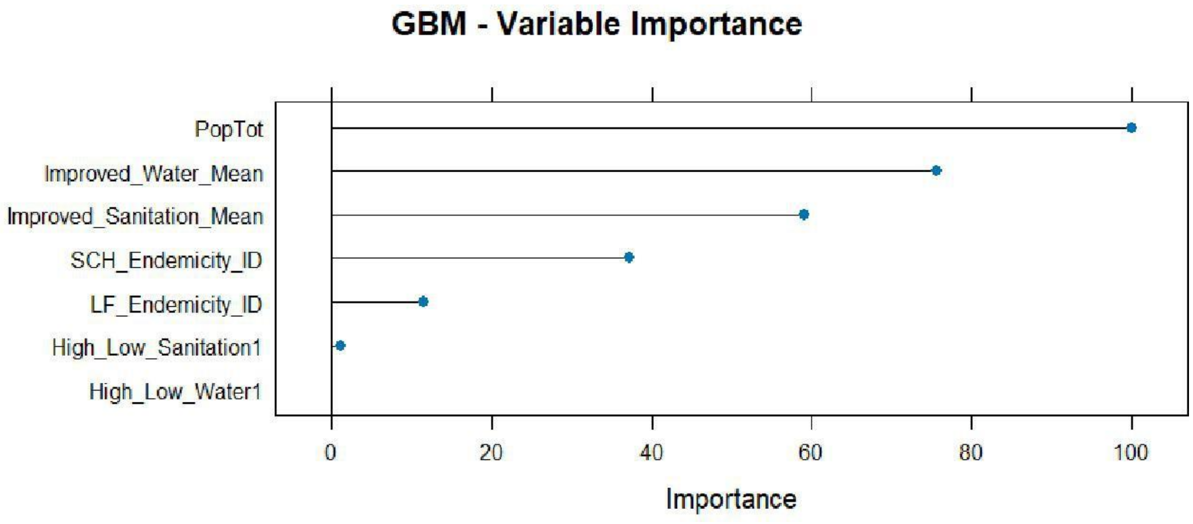

Figure 13: GBM variable importance

3.5.2 GBM performance on the test set

In Table 6, the model correctly predicted 61.4% of the cases. The p −value (0.1762) was not significant as compared to the NIR (54.39%). The model is good at identifying high cases (se nsitivity =77.42%). The kappa (0.2023), showed a slight agreement with the results. The mo del performed poorly in identifying low cases (specificity = 42.31%). A graph of sensitivity versus specificity quantifying the area under the ROC curve is shown in Figure 14.

Table 6: Performance metrics of the GBM model

Metric	Value
Accuracy	0.6140
95% CI	0.4757, 0.74
No Information Rate (NIR)	0.5439
P value	0.1762
Kappa	0.2023
Sensitivity	0.7742
Specificity	0.4231
Positive predicted	0.6154
Negative predicted	0.6111
Prevalence	0.5439
Detection rate	0.4211
Detection prevalence	0.6842
Balanced accuracy	0.5986

3.5.3 Confusion matrix of the GBM model

In equation (2), the model correctly predicted 24 (TP) and 11 (TN) accounting for 61.40% (positive predicted) accuracy where TP (True positives), FP (False positive), FN (False Negative) and TN (True Negatives).

$$\begin{pmatrix} 24(TP) & 15(FN) \\ 7(FP) & 11(TN) \end{pmatrix} \quad \ldots\ldots\ldots\ldots\ldots\ldots\ldots\ldots\ldots\ldots\ldots\ldots \quad (2)$$

Figure 14 reveals that the GBM model achieved an AUC of 0.620, indicating moderate classi fication performance. While this score is above the threshold of random guessing (AUC = 0.5), it suggests that the model's ability to distinguish between classes is modest and could benef it from further optimization or additional features.

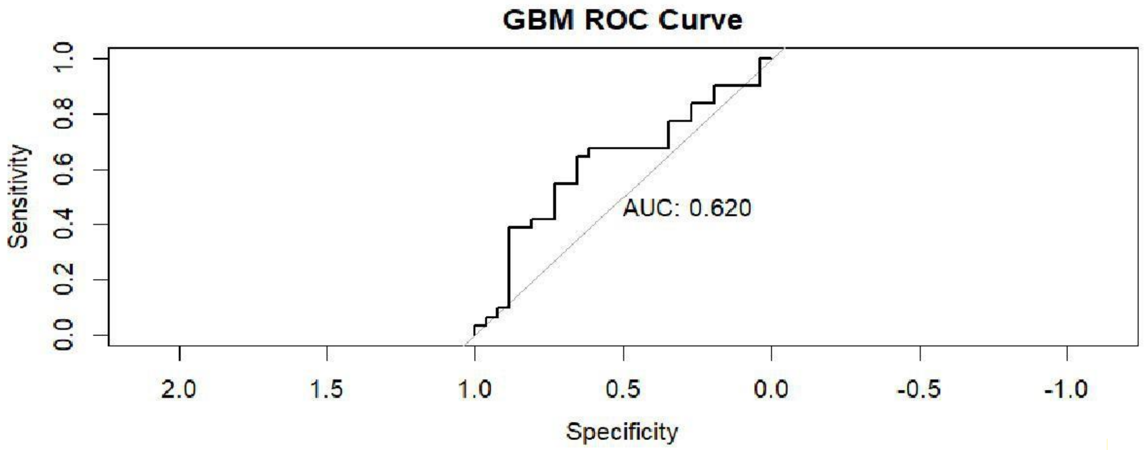

Figure 14: ROC curve of the GBM model

3.6 The XGBoost Model

The XGBoost model was evaluated using the tuning hyper parameters, ROC curve, variable i mportance features and the confusion matrix. The tuning parameter, γ ' was held constant at a value of 0. Tuning parameter 'min_child_weight' was held constant at a value of 1. ROC was used to select the optimal model using the largest value. The final values used for the model were nrounds = 100, max_depth =1, eta =0.4, gamma= 0, colsample_bytree = 0.6, min_child _weight =1 and subsample = 0.875. The key findings and metrics of the XGBoost model are as indicated in Figures 14, 15, 16 and Table 7. The confusion matrix is shown in equation (3)

3.6.1 XGBoost model illustration

XGBoost is a powerful, scalable machine learning algorithm based on gradient boosting. It builds an ensemble of decision trees sequentially, where each new tree tries to correct the errors of the previous ones [26]. Figure 15 shows the illustration of XGBoost model with the decision trees using hyper parameters.

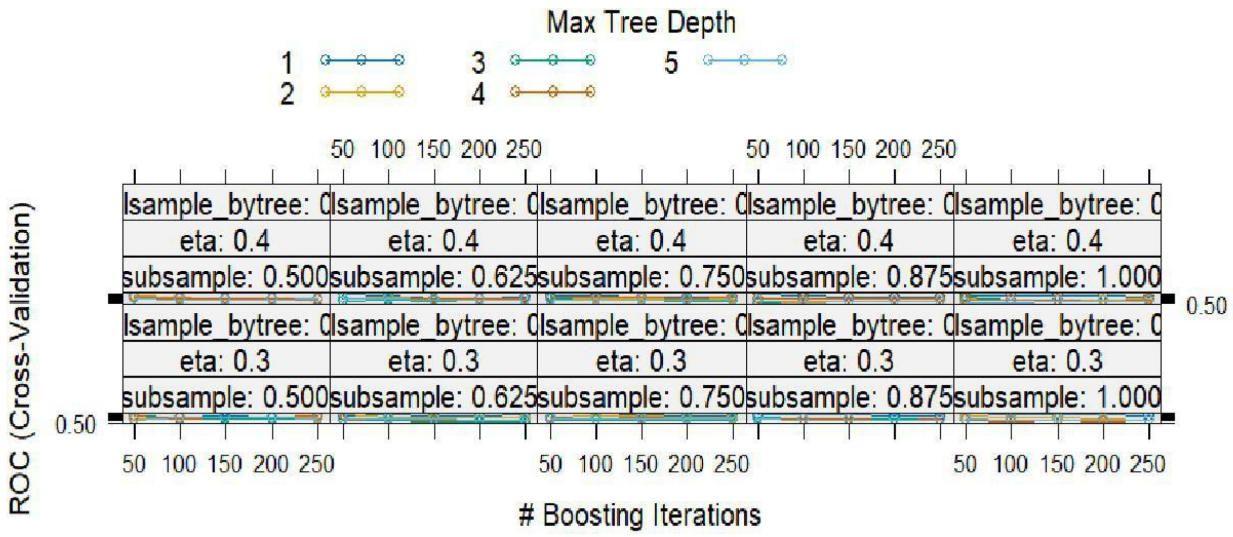

Figure 15: XGBoost model illustration

3.6.2 XGBoost model ROC curve

In Figure 16, the XGBoost model attained an AUC of 0.6560. The XGBoost model ROC curve was used to evaluate the relationship between the true positive rates (sensitivity) and the false positive rate (specificity).

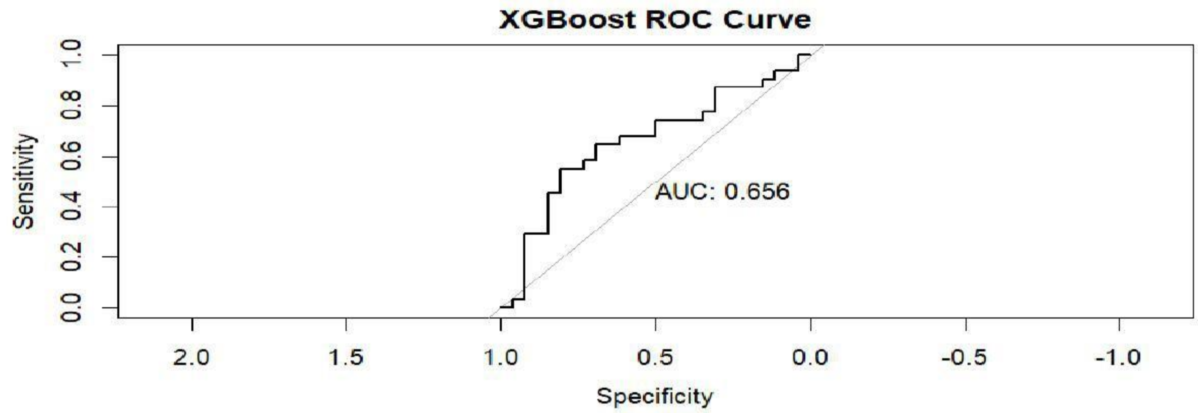

Figure 16: XGBoost ROC curve

3.6.3 XGBoost Variable importance features

The feature importance technique was applied in the XGBoost model to rank how much each feature (input variable) contributed to the predictive power of a model. As shown in Figure 17, the improved water mean (WASH variable) and the population total were the most important variables in predicting the disease co-endemicity under the XGBoost model.

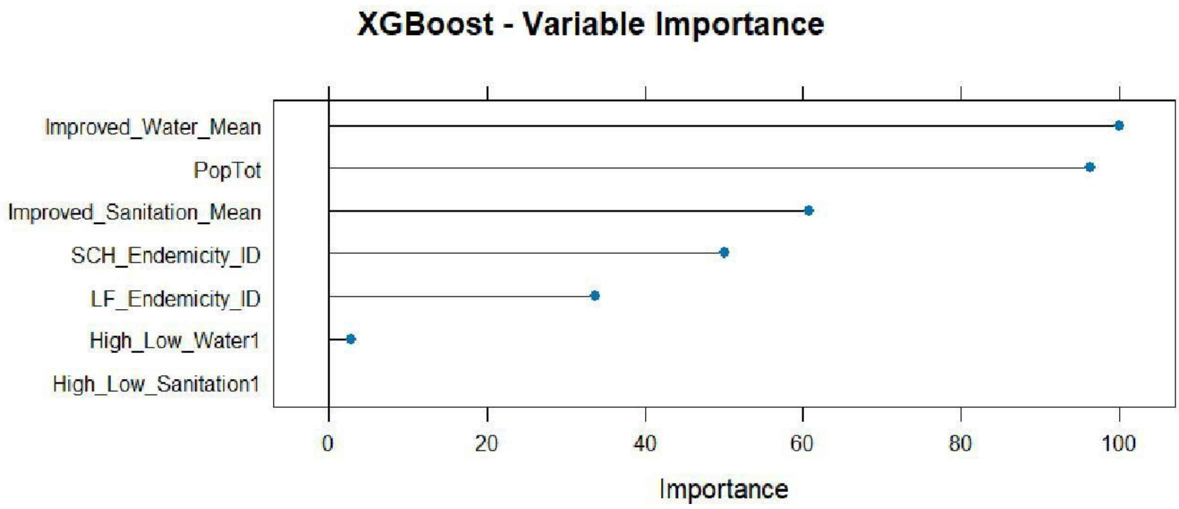

Figure 17: XGBoost variables importance

3.6.4 Confusion matrix and metrics

The confusion matrix of the XGBoost model is shown in equation (3) and the overall performance metrics are shown in Table 7.

Table 7: XGBoost performance metrics

Metric	Value
Accuracy	0.5965
95% CI	0.4582,0.7244
NIR	0.5439
P value	0.2540
Kappa	0.1687
Sensitivity	0.7419
Specificity	0.4231
Positive predicted	0.6053
Negative predicted	0.5789
Prevalence	0.5439
Detection rate	0.4035
Detection prevalence	0.6667
Balanced accuracy	0.5825

$$\begin{pmatrix} 23(TP) & 15(FN) \\ 8(FP) & 11(TN) \end{pmatrix} \quad \ldots\ldots\ldots\ldots\ldots\ldots\ldots\ldots\ldots\ldots\ldots\ldots\ldots\ldots\ldots \quad (3)$$

As shown in Table 7, the model's p-value (0.2540) shows that it isn't significant in the predictions. Based on the overall performance, the sensitivity (74.19%), specificity (42.31%) and accuracy (59.65%), shows that the model is reliable in detecting high cases, poor at identifying low cases and moderate in the prediction task respectively.

3.6.5 Models comparison

A comprehensive comparison of all the three models (RF, GBM and XGBoost) shown in Figure 18. The RF model (AUC=0.703) emerged the highest among the three while GBM model (AUC=0.62) was the lowest. The XGBoost (AUC=O.656) model was the middle in performance.

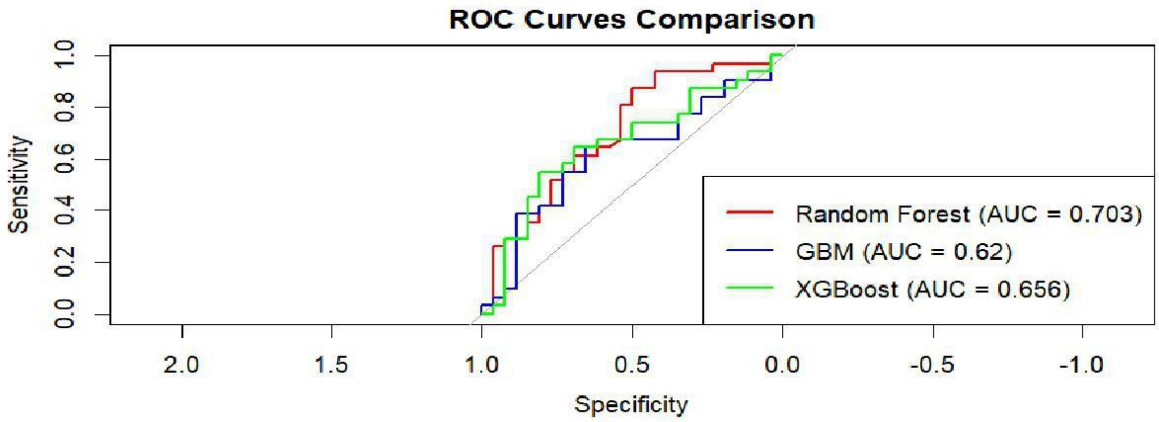

Figure 18: Models comparison

In Table 8, the models performance statistics based on ROC curves are shown. The RF model was the strongest with a maximum AUC of 0.775 and a mean of 0.5979. The XGBoost unveiled a competitive mean of 0.6167.

Table 8: Models performance based on the ROC curves

ROC	Min	Median	Mean	Maximum
RF	0.5128	0.5582	0.5979	0.7752
GBM	0.4809	0.5627	0.5616	0.6038
XGBoost	0.5273	0.6026	0.6167	0.6857

3.6.4 Overall Models comparison and performance

An overall performance of all the models based on the key performance metrics (Accuracy, S ensitivity, specificity, kappa and 95% confidence interval is shown in table 9.

Table 9: Models performance based on selected metrics.

Metric	RF	GBM	XGBoost
ROC AUC	0.7030	0.6200	0.6560
Accuracy	0.6842	0.6140	0.5965
Sensitivity	0.8387	0.7742	0.7419
Specificity	0.5000	0.4231	0.4231
Kappa	0.3473	0.2023	0.1687
95% CI	0.5476,0.8009	0.4757,0.7400	0.4582,0.7244

With a value of 0.7030, RF obtained the highest Area under the Receiver Operating Characteristic Curve (ROC AUC), demonstrating the best ability to distinguish between the positive and negative classes. Significantly better than the performance of the other two models (Kappa of 0.2023 for GBM and 0.1687 for XGBoost), this superior discrimination translated into a noteworthy Accuracy of 0.6842 and a Kappa statistic of 0.3473, suggesting moderate agreement beyond chance. With ROC AUC values of 0.6200 and 0.6560, respectively, the GBM and XGBoost models performed similarly, falling short of the RF approach's predictive power.

4. Discussion

The Expanded Special Project for the Elimination of Neglected Tropical Diseases (ESPEN) is collaborating directly with the ministries and other stakeholders of the Neglected Tropical Diseases (NTDs) to amplify the impact and elimination initiatives. In Africa, the total population which required preventive chemotherapy reduced from 592 million in 2016 to 588 million in 2019. This positive response was as a result of surveillance and improvement in research. Two countries namely Togo and Malawi were validated for completely eliminating lymphatic filariasis (LF) in 2017 and 2020 respectively. In Kenya, elimination and control of

NTDs has been prioritized. The NTDs disproportionately affect the most vulnerable, marginalized people in the poorest, most remote communities around the world.

The exploratory data analysis showed that all the Kenya's regions were at risk of NTDs. The endemicty levels of the three diseases (STH, LF and SCH) showed moderate to high prevalence. In terms of distribution and area coverage, the STH and SCH had higher distribution levels as compared to LF.

Our analysis demonstrates that machine learning models, particularly the RF model, effectively predict high STH prevalence areas in Kenya (AUC = 0.7030). The results of the RF model emerging as the most powerful for NTDs prediction and classification task aligns with those of [27], [28], [29]. Furthermore, RF algorithm is less prone to over fitting than the other models because it uses bootstrap aggregating (bagging) and random subspace sampling. It is less sensitive to hyper parameter tuning and its design inherently handles noise/outliers better. GBM and XGBoost, on the other hand, employ boosting, training trees one after the other, fixing the mistakes (residuals) of the previous tree. They are more prone to over fitting and capturing noise when applied to unseen (test) data, even though this reduces bias and frequently produces excellent performance on the training set. In contrast to GBM and particularly XGBoost, which frequently need extensive and meticulous hyper parameter tuning to prevent over fitting and attain optimal generalization performance, RF is frequently very effective with its default hyper parameter settings. The boosting models' propensity to over fit the training data was probably not adequately controlled by the default or basic tuning that was used.

The models identified improved sanitation, population total and water access as the strongest protective factors, consistent with global evidence linking WASH to STH reduction [19]. Notably, co - endemicity with LF and SCH emerged as significant predictors, suggesting overlapping transmission pathways that warrant integrated control strategies as evidenced in studies by [30], [31].

Spatial analysis revealed persistent hotspots in Eastern and North eastern counties, aligning with historical prevalence patterns [32] . These regions also reported lower WASH coverage, reinforcing the need for infrastructure investments alongside MDA. Conversely, Central and Nairobi counties exhibited lower STH burden, likely due to better urban sanitation systems. The study findings further recommends that Sanitation campaigns in schools and communities could disrupt STH transmission more effectively than standalone deworming.

Our findings concur with studies linking poor WASH to STH risk [7] but extend prior work by quantifying variable importance using ML. For instance, improved sanitation outweighed population density, suggesting that WASH interventions may yield greater impact than demographic factors alone. This contrasts with earlier regression-based models that emphasized population density highlighting ML's ability to capture complex interactions.

5. Conclusion

The study has demonstrated that machine learning methodology has the potential of classifying and predicting NTDs in Kenya with a key focus on the co-endemicity of STH, SCH and LF. Additionally, integration of the WASH variables and population data with the machine learning models provided insights and knowledge on the risk factors and the main hotspots in Kenya. Furthermore, the key findings indicated that RF model and the XGBoost outperformed the

GBM model in both the prediction and classification tasks. In the overall performance, the RF outperformed all the other models.

The exploratory analysis showed that the main co-endemic zones (Eastern and North eastern) have access to poor infrastructure and harsh climatic conditions hence underscoring the resistance of NTDs in those regions. In general, the study re-affirms that machine learning techniques are not only valuable tools for risk prediction but also a strategic enabler for targeted interventions, resource allocation, and proactive NTDs surveillance, especially in data-limited settings.

6. Recommendations

This study recommends the use of real-time machine learning dashboards for dynamic risk mapping and timely interventions. The study further recommends the cost-effectiveness analyses of water and sanitation versus mass drug administration strategies for maximum optimization. The Integration with climate data to forecast environmental transmission risks of the NTDs hence promoting one heath approach to disease control and mitigation measures. The high risk counties should be prioritized for mass drug administration procedures and other health programs to curb disease severity and spread.

Future machine learning models should prioritize interpretability and explain ability to increase trust and adoption among policymakers, allowing them to clearly see how variables and risk factors drive NTDs risk. Training programs and collaborations with local universities and health ministries should be strengthened to build capacity in machine learning, artificial intelligence, geospatial and geo statistical approaches and evidence-based NTDs control.

7. Study limitations

The study provided important knowledge on the applications of machine learning in NTDs modelling, however, there were some notable limitations. Firstly, comprehensive data was not available and some areas were faced with comparability of prevalence. There were limited social and demographic co-variates which could influence disease transmissions.

8. Declarations

Ethics approval and consent to participate

This dataset is publicly available via the link https://espen.afro.who.int/; and does not require ethical approvals for the preparation of the manuscript.

Consent for publication

All authors have approved the manuscript for submission

Availability of data and material

The data used in this study is publicly available via the link https://espen.afro.who.int/. Electronic data capture, with standardized data collection forms, and automatic encrypted data upload to a secure central server. Form testing with specialists and stakeholders is responsible for conducting the surveys to ensure the form will collect quality data and adapted to the specificities of the diseases.

Competing interests

The authors declare no competing interests

Funding

The study did not receive any specific funding

Authors' contributions

Conceptualization – DM, NN
Data curation – DM, NN
Formal analysis - DM
Investigation- NN
Methodology – DM, NN
Software- DM
Supervision - NN
Validation - DM
Visualization - DM
Writing – Original draft – DM, NN
Writing – review and editing - DM

Acknowledgements

The authors extend their appreciation to their universities for supporting their research work.

References

[1] "Prevalence and distribution pattern of malaria and soil-transmitted helminth co-endemicity in sub-Saharan Africa, 2000–2018: A geospatial analysis | PLOS Neglected Tropical Diseases." Accessed: May 31, 2025. [Online]. Available: https://journals.plos.org/plosntds/article?id=10.1371/journal.pntd.0010321

[2] "The global burden of disease study 2013: What does it mean for the NTDs? | PLOS Neglected Tropical Diseases." Accessed: Jul. 03, 2025. [Online]. Available: https://journals.plos.org/plosntds/article?id=10.1371/journal.pntd.0005424

[3] "ESPEN | Expanded Special Project for Elimination Neglected Tropical Diseases." Accessed: May 31, 2025. [Online]. Available: https://espen.afro.who.int/

[4] "Historical overview and geographical distribution of neglected tropical diseases amenable to preventive chemotherapy in the Republic of the Congo: A systematic review | PLOS Neglected Tropical Diseases." Accessed: Jul. 03, 2025. [Online]. Available: https://journals.plos.org/plosntds/article?id=10.1371/journal.pntd.0010560

[5] "'Our interventions are still here to support communities during the pandemic': Resuming mass drug administration for neglected tropical diseases after COVID-19 implementation delays | PLOS Neglected Tropical Diseases." Accessed: Jul. 03, 2025. [Online]. Available: https://journals.plos.org/plosntds/article?id=10.1371/journal.pntd.0011368

[6] O. T. Oyeyemi, O. Ogundahunsi, M. Schunk, R. G. Fatem, and L. M. Shollenberger, "Neglected tropical disease (NTD) diagnostics: current development and operations to advance control," *Pathog Glob Health*, vol. 118, no. 1, pp. 1–24, doi: 10.1080/20477724.2023.2272095.

[7] A. Echazú *et al.*, "Effect of Poor Access to Water and Sanitation As Risk Factors for Soil-Transmitted Helminth Infection: Selectiveness by the Infective Route," *PLOS Neglected Tropical Diseases*, vol. 9, no. 9, p. e0004111, Sep. 2015, doi: 10.1371/journal.pntd.0004111.

[8] "Lymphatic Filariasis | SpringerLink." Accessed: Jun. 02, 2025. [Online]. Available: https://link.springer.com/book/10.1007/978-981-10-2257-9

[9] E. M. M. da Rocha, G. Fontes, and J. P. Ehrenberg, "Lymphatic Filariasis," in *Arthropod Borne Diseases*, C. B. Marcondes, Ed., Cham: Springer International Publishing, 2017, pp. 369–381. doi: 10.1007/978-3-319-13884-8_24.

[10] W. Melrose and J. M. Goldsmid, "Infections of the lymphatic system".

[11] "The psychosocial and emotional burden of lymphatic filariasis: A systematic review | PLOS Neglected Tropical Diseases." Accessed: Jun. 02, 2025. [Online]. Available: https://journals.plos.org/plosntds/article?id=10.1371/journal.pntd.0013073

[12] "Effect of Sanitation on Soil-Transmitted Helminth Infection: Systematic Review and Meta-Analysis | PLOS Medicine." Accessed: Jun. 02, 2025. [Online]. Available: https://journals.plos.org/plosmedicine/article?id=10.1371/journal.pmed.1001162

[13] "Prevalence, risk factors, challenges, and the currently available diagnostic tools for the determination of helminths infections in human - Muhammad Riaz, Nosheen Aslam, Rida Zainab, Aziz-Ur-Rehman, Ghulam Rasool, Muhammad Irfan Ullah, Muhammad Daniyal, Muhammad Akram, 2020." Accessed: Jul. 03, 2025. [Online]. Available: https://journals.sagepub.com/doi/full/10.1177/2058739220959915

[14] "Challenges and opportunities for control and elimination of soil-transmitted helminth infection beyond 2020 | PLOS Neglected Tropical Diseases." Accessed: Jul. 03, 2025. [Online]. Available: https://journals.plos.org/plosntds/article?id=10.1371/journal.pntd.0007201

[15] “Soil-Transmitted Helminths and Anaemia: A Neglected Association Outside the Tropics.” Accessed: Jun. 02, 2025. [Online]. Available: https://www.mdpi.com/2076-2607/10/5/1027

[16] D. U. Olveda *et al.*, “Bilharzia: Pathology, Diagnosis, Management and Control,” *Trop Med Surg*, vol. 1, no. 4, p. 135, Aug. 2013, doi: 10.4172/2329-9088.1000135.

[17] M. L. Nelwan, “Schistosomiasis: Life Cycle, Diagnosis, and Control,” *Current Therapeutic Research*, vol. 91, pp. 5–9, Jan. 2019, doi: 10.1016/j.curtheres.2019.06.001.

[18] “Evaluation of the Health-related Quality of Life of Children in Schistosoma haematobium-endemic Communities in Kenya: A Cross-sectional Study | PLOS Neglected Tropical Diseases.” Accessed: Jul. 03, 2025. [Online]. Available: https://journals.plos.org/plosntds/article?id=10.1371/journal.pntd.0002106

[19] “Water, Sanitation, Hygiene, and Soil-Transmitted Helminth Infection: A Systematic Review and Meta-Analysis | PLOS Medicine.” Accessed: May 31, 2025. [Online]. Available: https://journals.plos.org/plosmedicine/article?id=10.1371/journal.pmed.1001620

[20] “Geographical Inequalities in Use of Improved Drinking Water Supply and Sanitation across Sub-Saharan Africa: Mapping and Spatial Analysis of Cross-sectional Survey Data | PLOS Medicine.” Accessed: May 31, 2025. [Online]. Available: https://journals.plos.org/plosmedicine/article?id=10.1371/journal.pmed.1001626

[21] “Machine learning, artificial intelligence, and data science breaking into drug design and neglected diseases - Peña-Guerrero - 2021 - WIREs Computational Molecular Science - Wiley Online Library.” Accessed: May 31, 2025. [Online]. Available: https://wires.onlinelibrary.wiley.com/doi/abs/10.1002/wcms.1513

[22] “Prevalence and risk factors of schistosomiasis and hookworm infection in seasonal transmission settings in northern Côte d’Ivoire: A cross-sectional study | PLOS Neglected Tropical Diseases.” Accessed: May 31, 2025. [Online]. Available: https://journals.plos.org/plosntds/article?id=10.1371/journal.pntd.0011487

[23] “Applications of machine learning techniques to predict filariasis using socio-economic factors | Epidemiology & Infection | Cambridge Core.” Accessed: May 31, 2025. [Online]. Available: https://www.cambridge.org/core/journals/epidemiology-and-infection/article/applications-of-machine-learning-techniques-to-predict-filariasis-using-socioeconomic-factors/61786B469CCCF578C24E106F42813FE2

[24] O. Augustine Omondi, “Analysis of Meteorological Drought in North Eastern Province of Kenya,” *Journal of earth science and climate change*, vol. 5, Sep. 2014.

[25] “Assessing the predictive capability of ensemble tree methods for landslide susceptibility mapping using XGBoost, gradient boosting machine, and RF | Discover Applied Sciences.” Accessed: Jun. 02, 2025. [Online]. Available: https://link.springer.com/article/10.1007/s42452-020-3060-1

[26] T. Chen and C. Guestrin, “XGBoost: A Scalable Tree Boosting System,” in *Proceedings of the 22nd ACM SIGKDD International Conference on Knowledge Discovery and Data Mining*, in KDD ’16. New York, NY, USA: Association for Computing Machinery, Aug. 2016, pp. 785–794. doi: 10.1145/2939672.2939785.

[27] A. W. Oehm *et al.*, “RF classification as a tool in epidemiological modelling: Identification of farm-specific characteristics relevant for the occurrence of Fasciola hepatica on German dairy farms,” *PLOS ONE*, vol. 18, no. 12, p. e0296093, Dec. 2023, doi: 10.1371/journal.pone.0296093.

[28] G. Machado, M. R. Mendoza, and L. G. Corbellini, “What variables are important in predicting bovine viral diarrhea virus? A RF approach,” *Vet Res*, vol. 46, no. 1, p. 85, Jul. 2015, doi: 10.1186/s13567-015-0219-7.

[29] A. Zafar *et al.*, “Machine learning-based risk factor analysis and prevalence prediction of intestinal parasitic infections using epidemiological survey data,” *PLOS Neglected Tropical Diseases*, vol. 16, no. 6, p. e0010517, Jun. 2022, doi: 10.1371/journal.pntd.0010517.

[30] N. Midzi *et al.*, “Distribution of Schistosomiasis and Soil Transmitted Helminthiasis in Zimbabwe: Towards a National Plan of Action for Control and Elimination,” *PLOS Neglected Tropical Diseases*, vol. 8, no. 8, p. e3014, Aug. 2014, doi: 10.1371/journal.pntd.0003014.

[31] G. T. Leta *et al.*, “National mapping of soil-transmitted helminth and schistosome infections in Ethiopia,” *Parasites Vectors*, vol. 13, no. 1, p. 437, Sep. 2020, doi: 10.1186/s13071-020-04317-6.

[32] R. W. Kihoro *et al.*, “Epidemiology of soil-transmitted helminthiasis among school-aged children in pastoralist communities of Kenya: A cross-sectional study,” *PLOS ONE*, vol. 19, no. 5, p. e0304266, May 2024, doi: 10.1371/journal.pone.0304266.

List of legends

Table 1: Creation of target variable.

Table 1 shows the key steps followed were the creation target variable, (co-endemicity class) whereby the disease endemicity status were combined into one categorical variable. One hot encoding was employed to convert categorical variables into binary outcomes for the machine learning

Table 2: Correlation matrix.

The correlation matrix shows the relationship between the target variable and the predictors. Table 2 unveils some important relationships between the disease endemicity status, population structure and the WASH variables.

Table 3: Model overall performance metrics and statistics of the RF model.

Table 3 indicates that in the overall model, the predictive power was low since the ROC (0.5979) < 0.7. The sensitivity (0.6600) clearly indicates that only 66% of high-risk areas will be correctly identified and 53% (specificity) of low-risk areas will be misclassified as high-risk.

Table 4: RF model key performance metrics.

Table 4 shows that in the prediction task, the RF model accurately predicted 68.42% of the disease cases. The wide confidence interval suggests a good variability in the model. The P - value (0.0219) was statistically significant and was better than random guessing (NIR = 54.39 %). The model’s sensitivity (83.87%) means that it was able to detect the actual cases and wa s able to minimize false negatives. Only 50% of the actual low cases were correctly identified (specificity). The mean of sensitivity and specificity (balanced accuracy was 0.6694).
The kappa, which is interpreted as a fair agreement between the variables was 0.3473.

Table 5: Resampling results across tuning parameters for the RF model.

Table 5 shows the results of resampling across the tuning parameters. The best model was identified using the ROC metric (0.5691) and had $n.trees = 150$ and a depth of 2. The highest sensitivity (0.7068) was observed at interaction depth 1 and had a specificity of 0.3065. The final values used for the model were n.trees = 50, interaction.depth = 2, shrinkage = 0.1 and n.minobsinnode = 10.

Table 6: Performance metrics of the GBM model.

In Table 6, the model correctly predicted 61.4% of the cases. The p $-$value (0.1762) was not significant as compared to the NIR (54.39%). The model is good at identifying high cases (sensitivity =77.42%). The kappa (0.2023), showed a slight agreement with the results. The model performed poorly in identifying low cases (specificity = 42.31%).

Table 7: XGBoost performance metrics.

Table 7 shows that the model's p-value (0.2540) wasn't significant in the predictions. Based on the overall performance, the sensitivity (74.19%), specificity (42.31%) and accuracy (59.65%), shows that the model is reliable in detecting high cases, poor at identifying low cases and moderate in the prediction task respectively.

Table 8: Models performance based on the ROC curves.

In Table 8, the models performance statistics based on ROC curves are shown. The RF model was the strongest with a maximum AUC of 0.775 and a mean of 0.5979. The XGBoost unveiled a competitive mean of 0.6167.

Table 9: Models performance based on selected metrics.

Table 9 shows an overall performance of all the models based on the key performance metrics (Accuracy, Sensitivity, specificity, kappa and 95% confidence interval).

Click here to access/download;Figure;fig1.docx

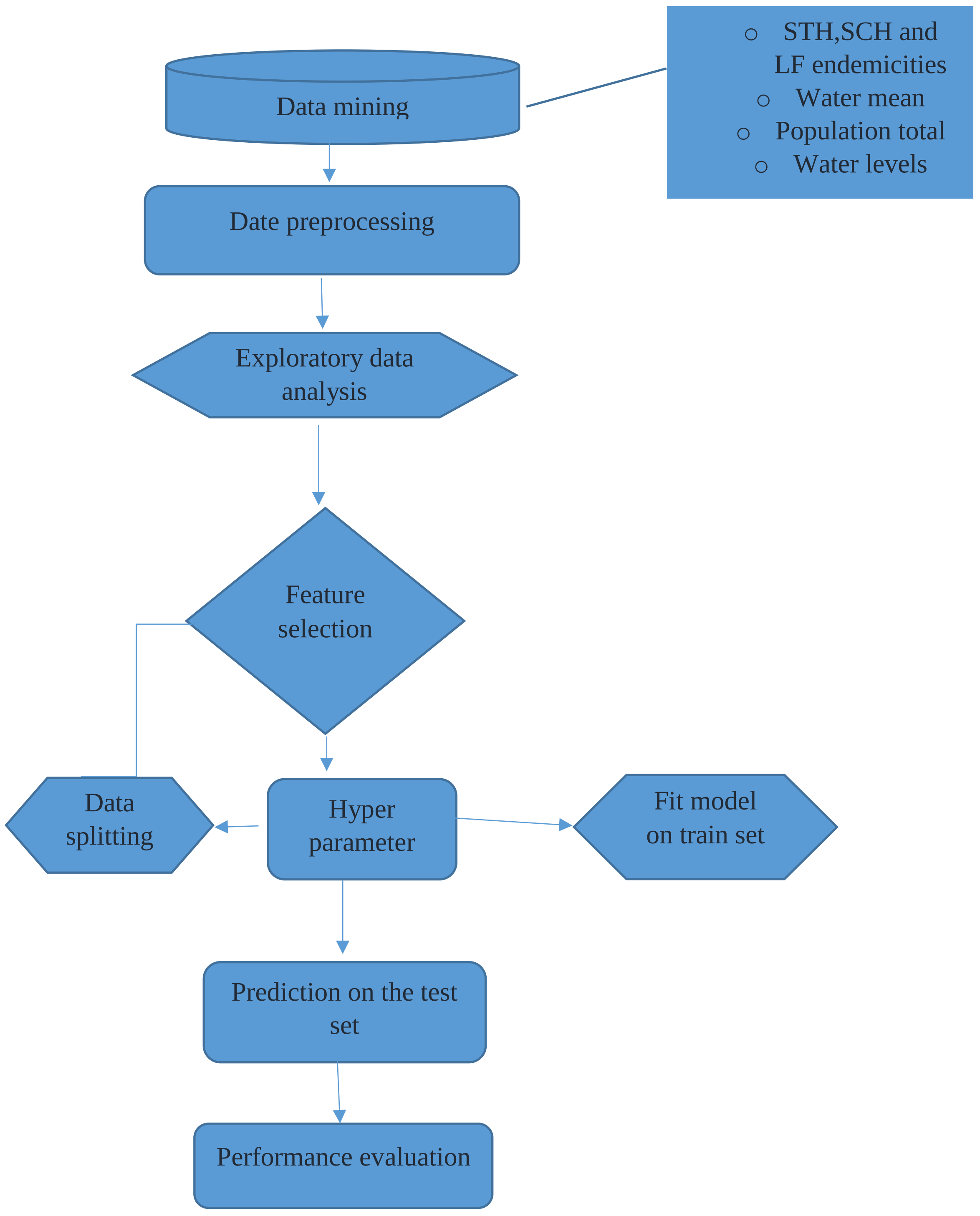

Figure 1: Flowchart for the proposed methodology

Click here to access/download;Figure;fig2.docx

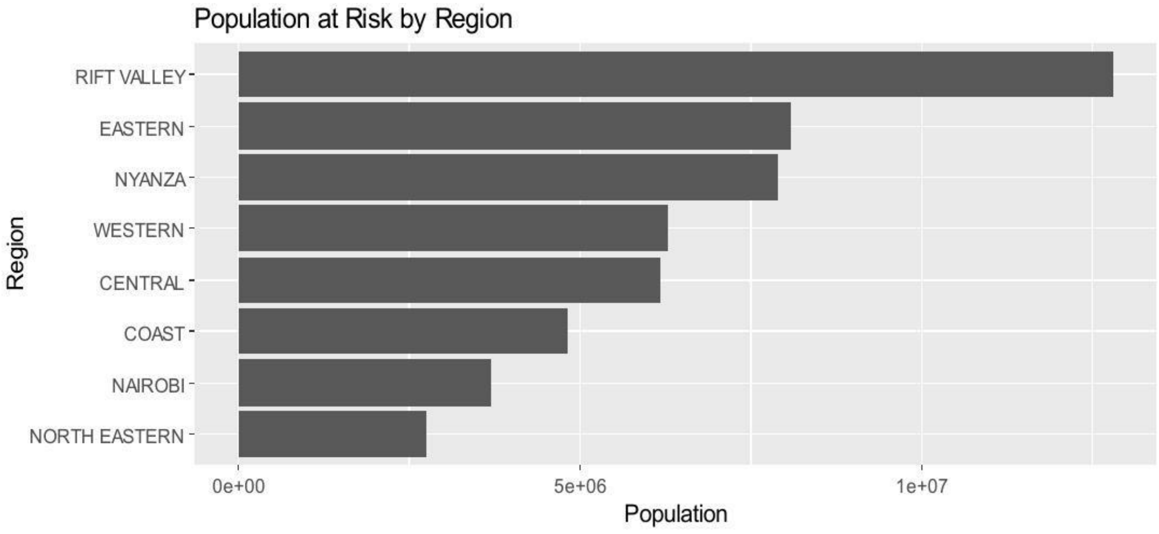

Figure 2: The population at risk of NTDs by region

Click here to access/download;Figure;fig3.docx

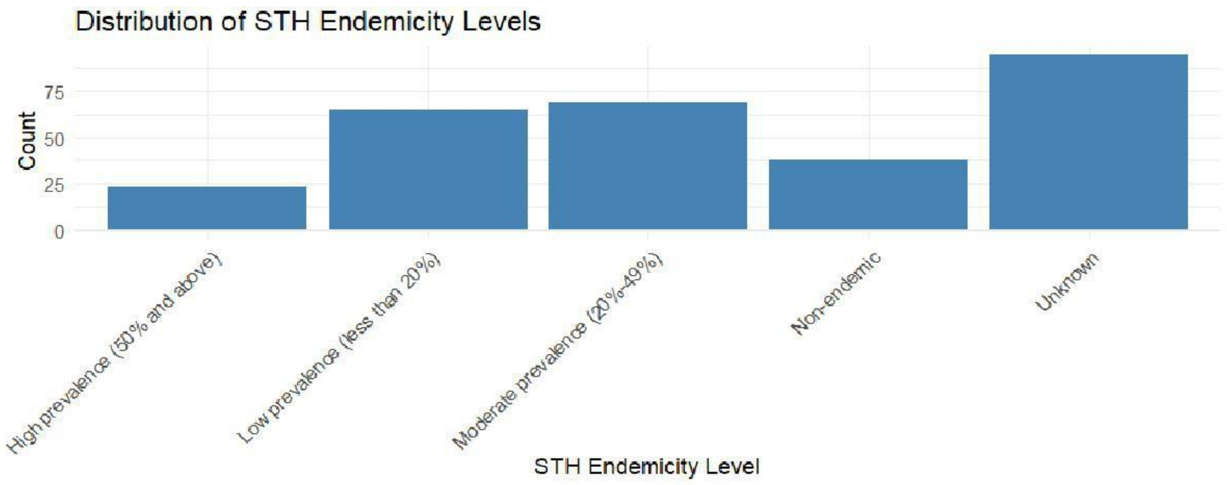

Figure 3: Distribution of STH endemicity levels

Click here to access/download;Figure;fig4.docx

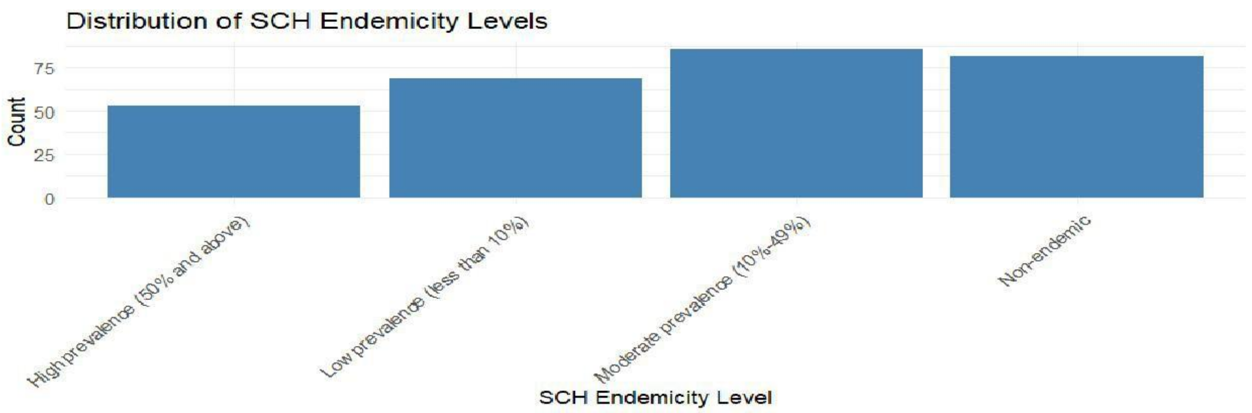

Figure 4: Distribution of SCH endemicity levels

Click here to access/download;Figure;fig5.docx

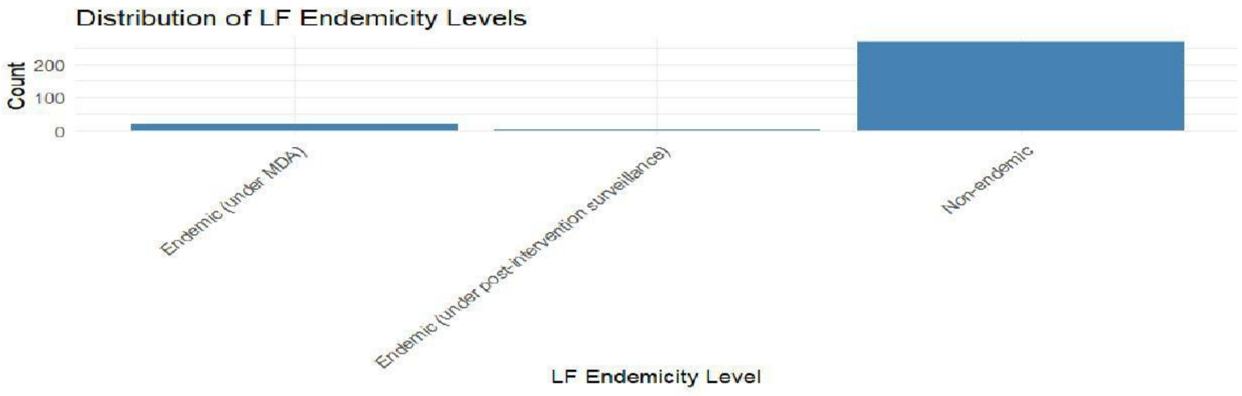

Figure 5: Distribution of LF endemicity levels

Click here to access/download;Figure;fig6.docx

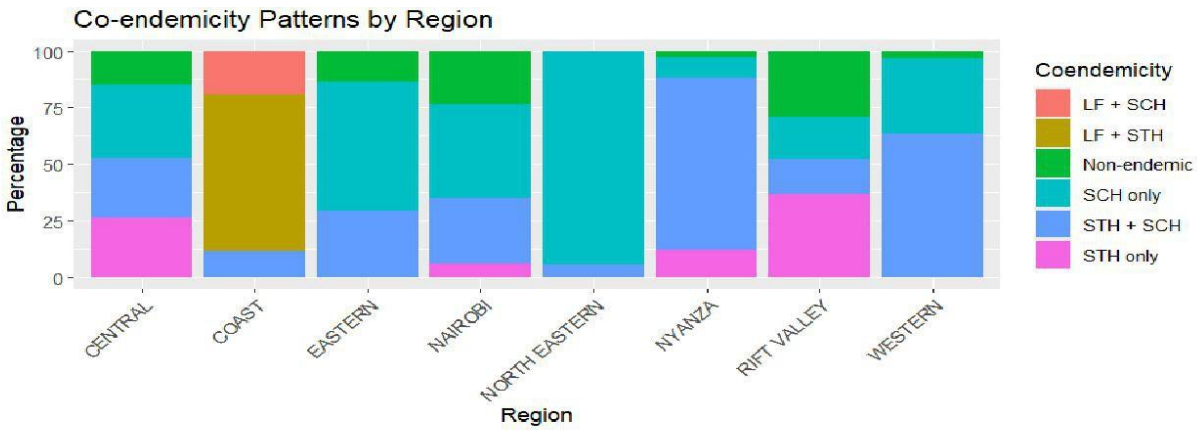

Figure 6: NTDs co-endemicity status by region

Click here to access/download;Figure;fig7.docx

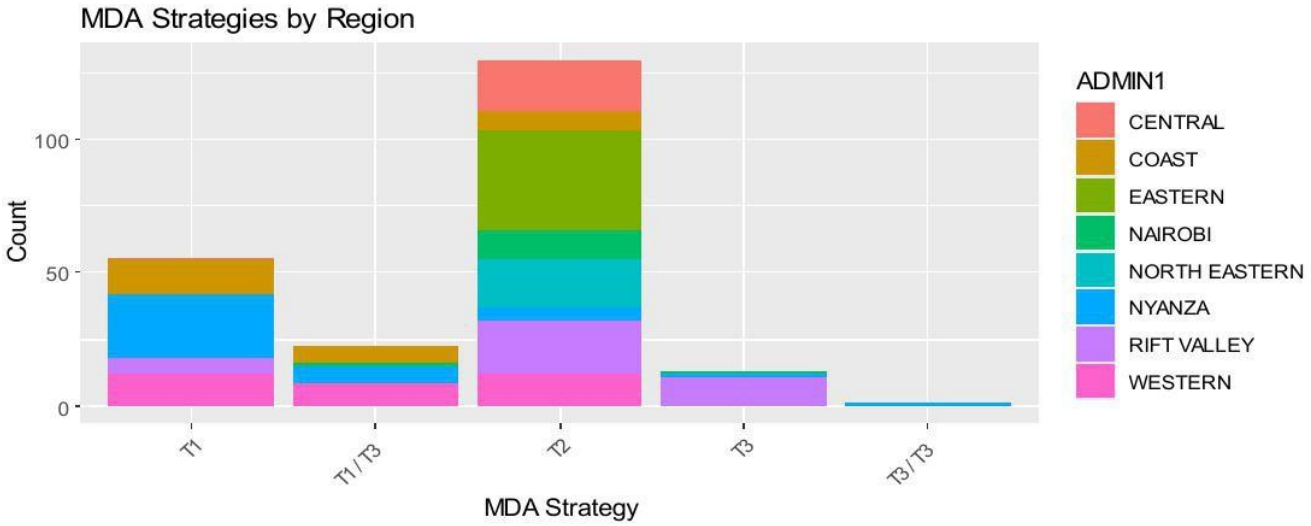

Figure 7: MDA strategies and usage by region

Click here to access/download;Figure;fig8.docx

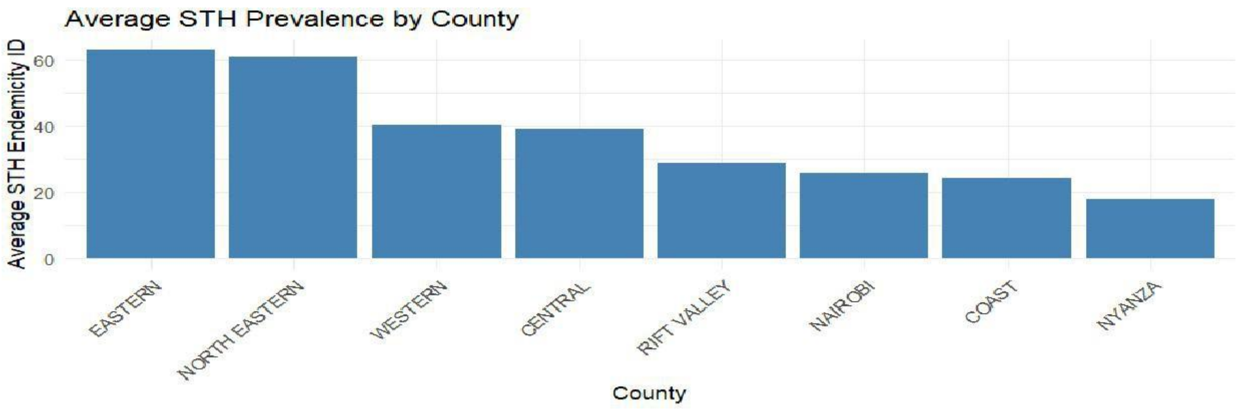

Figure 8: STH prevalence by region in Kenya

Click here to access/download;Figure;fig9.docx

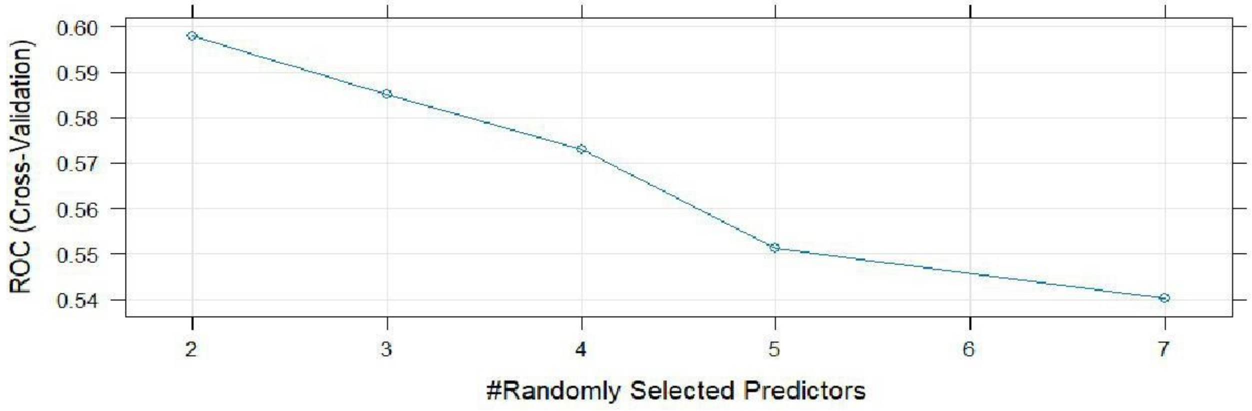

Figure 9: Random Forest plot

Click here to access/download;Figure;fig10.docx

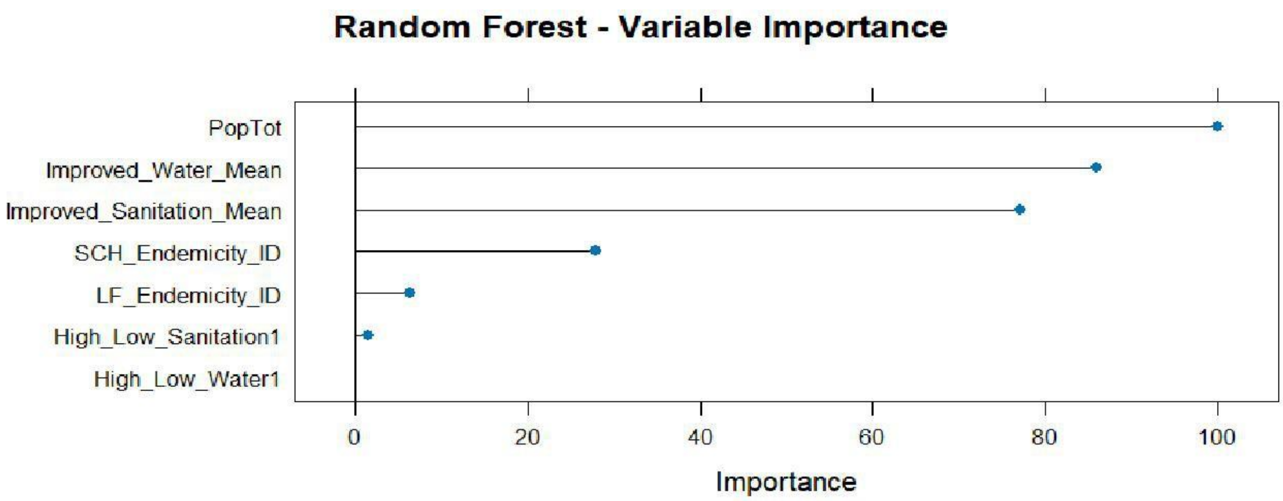

Figure 10: RF Variable importance

Click here to access/download;Figure;fig11.docx

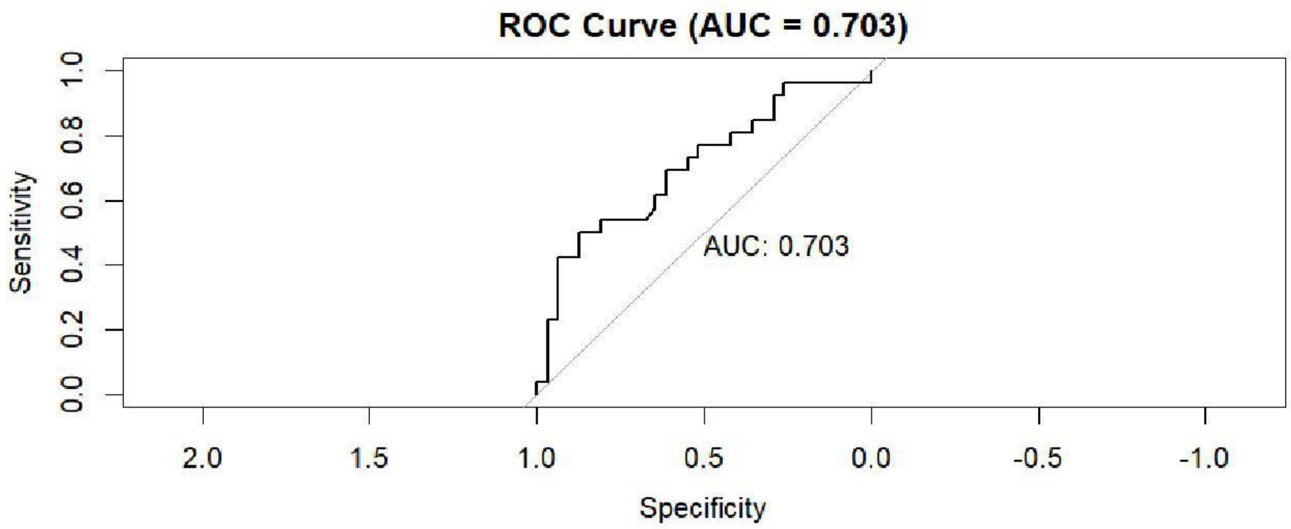

Figure 11: RF ROC AUC

Click here to access/download;Figure;fig12.docx

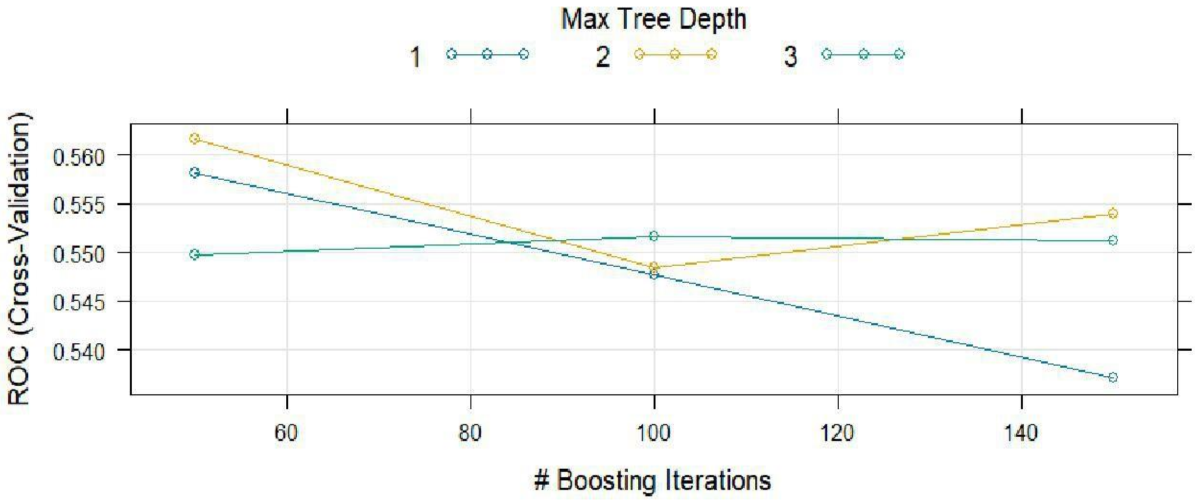

Figure 12: GBM model hyper parameter results

Click here to access/download;Figure;fig13.docx

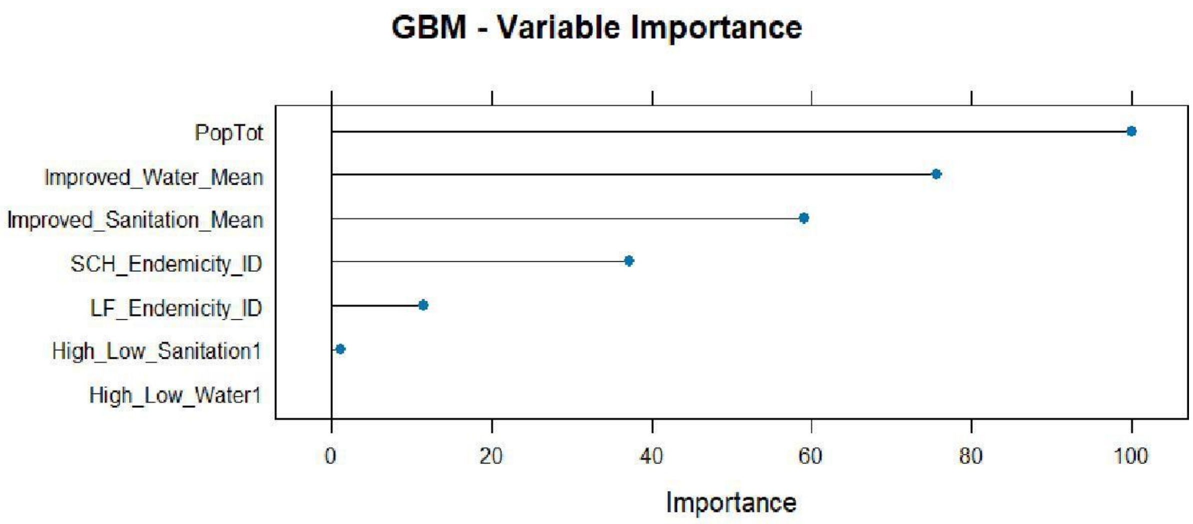

Figure 13: GBM variable importance

Click here to access/download;Figure;fig14.docx

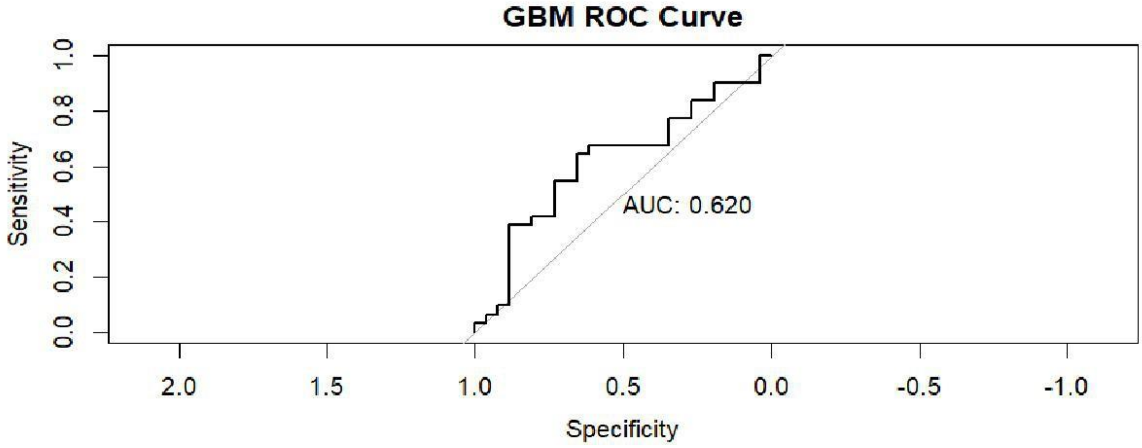

Figure 14: ROC curve of the GBM model

Click here to access/download;Figure;fig15.docx

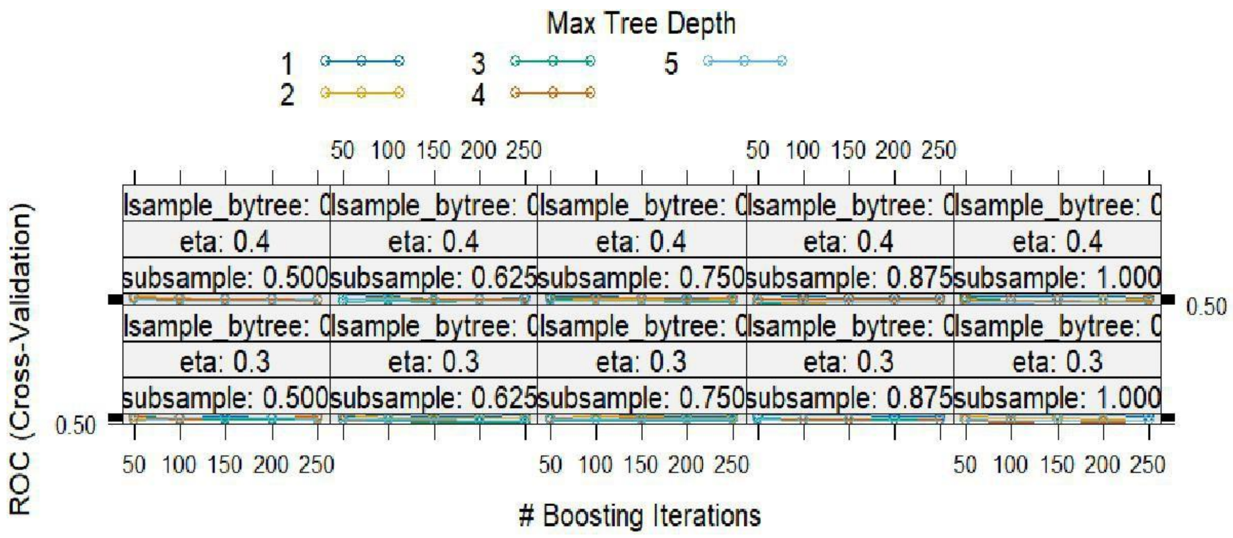

Figure 15: XGBoost model illustration

Click here to access/download;Figure;fig16.docx

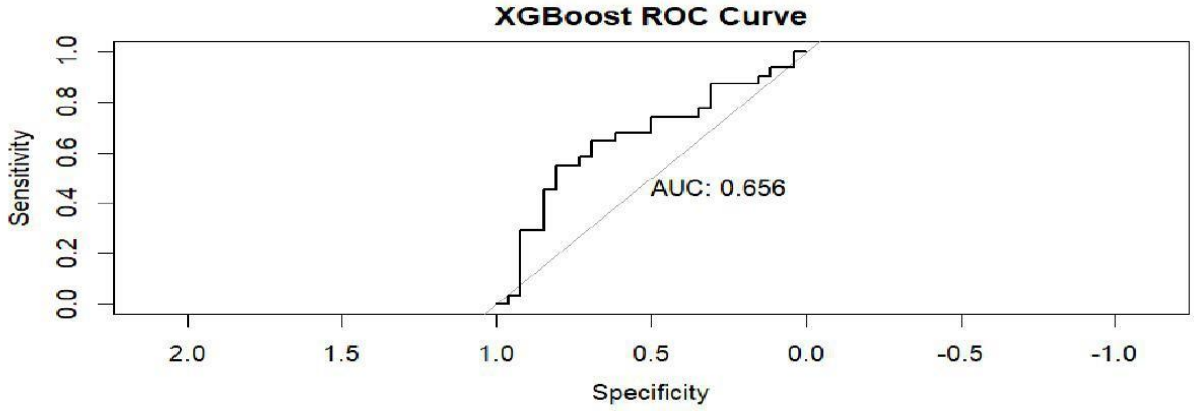

Figure 16: XGBoost ROC curve

Click here to access/download;Figure;fig17.docx

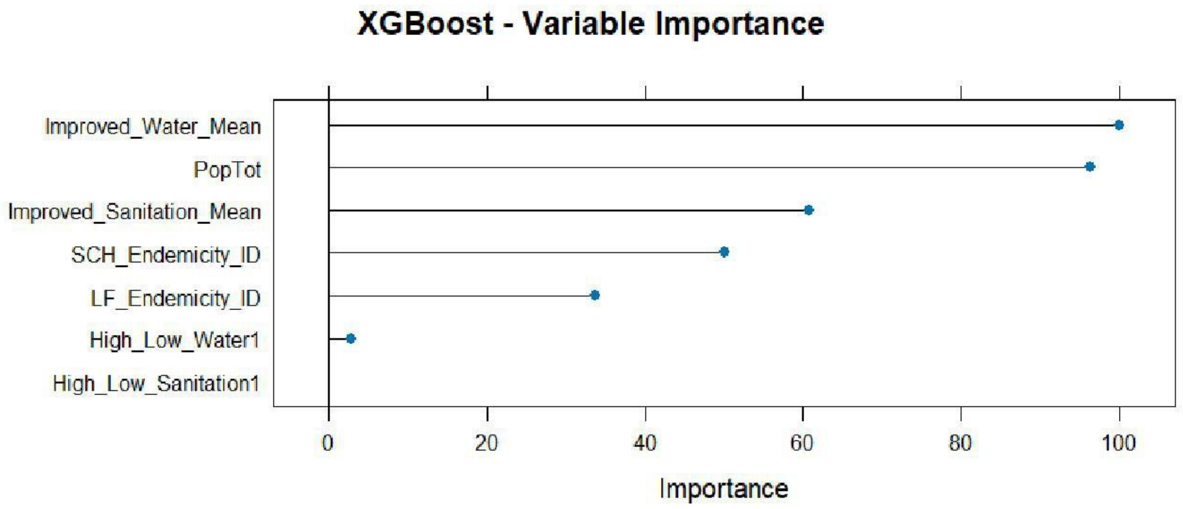

Figure 17: XGBoost variables importance

Click here to access/download;Figure;fig18.docx

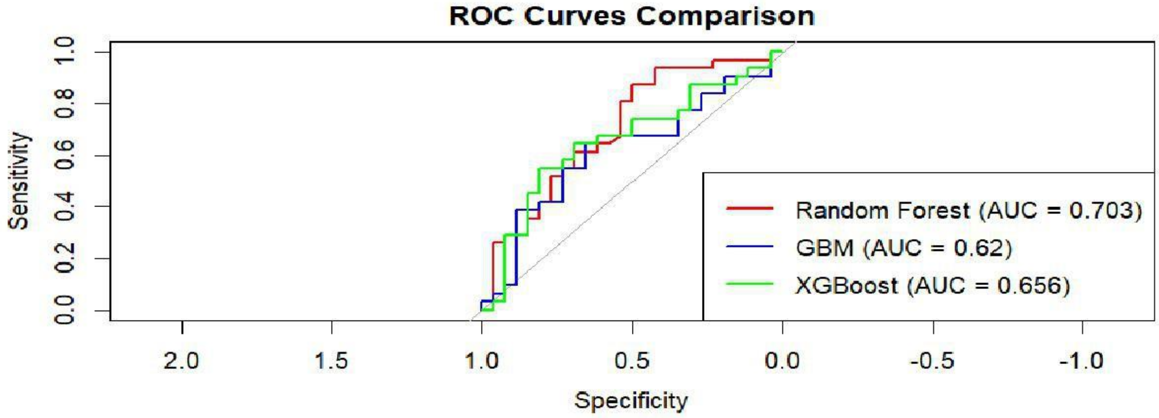

Figure 18: Models comparison

Click here to access/download
Supporting Information
data-coendemicity-KE-2022.xlsx

Advanced Machine Learning Approaches for Predicting Neglected Tropical Disease (NTD) Co-endemicity in Kenya: A Focus on Soil-Transmitted Helminthes, Schistosomiasis, and Lymphatic Filariasis.

Reviewer Comments:

Reviewer 1:

1. Model comparison was done only using AUC? Why not with other parameters?
   Response: We greatly appreciate the reviewer's suggestion. We have added a table (table 9) summarizing all the key classification metrics for RF, GBM, and XGBoost on the test set including Accuracy, kappa, sensitivity, specificity and confidence intervals.
   Line 578-592 as shown below:

   Table 9: Models performance based on selected metrics.

Metric	RF	GBM	XGBoost
ROC AUC	0.7030	0.6200	0.6560
Accuracy	0.6842	0.6140	0.5965
Sensitivity	0.8387	0.7742	0.7419
Specificity	0.5000	0.4231	0.4231
Kappa	0.3473	0.2023	0.1687
95% CI	0.5476,0.8009	0.4757,0.7400	0.4582,0.7244

   With a value of 0.7030, RF obtained the highest Area under the Receiver Operating Characteristic Curve (ROC AUC), demonstrating the best ability to distinguish between the positive and negative classes. Significantly better than the performance of the other two models (Kappa of 0.2023 for GBM and 0.1687 for XGBoost), this superior discrimination translated into a noteworthy Accuracy of 0.6842 and a Kappa statistic of 0.3473, suggesting moderate agreement beyond chance. With ROC AUC values of 0.6200 and 0.6560, respectively, the GBM and XGBoost models performed similarly, falling short of the RF approach's predictive power.

2. The comparison graph AUC is not scaled properly and the graph can be much improved.
   Response: Thank you for this great observation. We have fitted the graph to a proper scale and improved it accordingly. Additionally, we have used the PLOS's NAAS tool to improve all the figures in the entire manuscript.

3. Why you have chosen only three ML algorithms namely RF, GBM and XG boost? Any specific reasons?
   Response: Many thanks for this great concern. We have added a section in the methodology section (2.8) on "Algorithm Selection Justification" as shown below.
   Lines 272 – 279.

The study chose only three machine learning algorithms namely RF, GBM and XGBoost. Firstly, the three algorithms are tree-based ensemble methods known for robustness to non-linear and complex relationships which are common in disease data, handling mixed data types i.e., categorical, continuous, discrete and without extensive feature scaling and lastly providing feature importance Scores (key for interpretability in disease modelling context). The Random forest and XGBoost are the leading tree-based ensembles selected for their well-documented performance on structured data and their ability to capture non-linearities.

4. Why other classification algorithms are not chosen? Give Reasons.
Response: We appreciate this question. We have addressed this in no.3 above whereby the algorithms were chosen purposefully due to their robustness to nonlinear and complex data.

5. What is the reason RF is giving good results compared to other algorithms take for experiment?
Response: Many thanks for this great concern. We have included a paragraph in the discussion section explaining the "surprising" results of the RF giving better results XGBoost and GBM.
Lines 611-621 as shown below.
Firstly, RF is less prone to over fitting than the other models because it uses bootstrap aggregating (bagging) and random subspace sampling. It is less sensitive to hyper parameter tuning and its design inherently handles noise/outliers better. GBM and XGBoost, on the other hand, employ boosting, training trees one after the other, fixing the mistakes (residuals) of the previous tree. They are more prone to over fitting and capturing noise when applied to unseen (test) data, even though this reduces bias and frequently produces excellent performance on the training set. In contrast to GBM and particularly XGBoost, which frequently need extensive and meticulous hyper parameter tuning to prevent over fitting and attain optimal generalization performance, RF is frequently very effective with its default hyper parameter settings. The boosting models' propensity to over fit the training data was probably not adequately controlled by the default or basic tuning that was used.

6. Whether the classes in the dataset are uniformly distributed?
Response: Thank you for this insightful input. We have included the class imbalance technique in the methodology section (section 2.4) as shown below.
Lines 158 – 164;
Data cleaning was carried out in R software version 4.5.0. Missing values for the disease data were detected during cleaning and were replaced using the mean of the complete samples. Since the data was balanced (35:65), there was no need of re-sampling the minority class. The data was balanced to a moderate extent to perform the training process to build the co - endemicity prediction.

7. How many features are in the dataset? Whether you considered all? No discussion in this regard.
Response: We appreciate this comment. We have added a sub section on feature selection and preparation, number of features selected, number of features removed and the criteria used in the methodology section (2.4) as shown below.
Lines 159-165;
Data cleaning was carried out in R software version 4.5.0. The dataset contained 37 variables which were exclusively selected using the variable importance criterion. The missing values for the diseases data were detected during cleaning and were replaced using the mean of the complete samples. Since the data was balanced (35:65), there was no need of re-sampling the minority class. The data was balanced to a moderate extent to perform the training process to build the co - endemicity prediction. Sections 2.5 and 2.6 give more details on the variables used in the study.

8. No details about the size of the dataset?
Response: Thank you for this great question. We have added the details about the size of the dataset including the data size, the number of geographic units, the number of features, and the final data split in the methodology section as shown below;
Lines 137-143
In this study, secondary data analysis using the Expanded Special Project for Elimination of Neglected Tropical Diseases (ESPEN) in Kenya's co - endemicty data for the year 2022 was done. Kenya, on the east coast of Africa, is endemic for LF, Schistosomiasis and STH. The Ministry of Health implements a Strategic Plan for control of NTDs through the National NTD control program. This study analyzed data at both national and subnational levels (implementation units) including a total of 47 counties and 290 implementation units which was sufficient for modelling.

9. Why deep learning models are not preferred for the given problem?
Response: We appreciate this good question and concern. Deep Learning models (like CNNs or RNNs) were not used because they require a much larger dataset (millions of observations) to train effectively and lacks the interpretability (feature importance) crucial for this NTDs modeling task.

10. Details such as missing values, categorical variables and other details in exploratory data analysis are missing.
Response: We appreciate this addition. We have expanded the methodology section and added details on missing values, categorical variables and data cleaning.
Lines 159 - 165; 226 - 230.

11. Figure 1 need to be of high clarity. Currently it is not so. Please change. Highlight the area which is being focused in this research.

Response: We appreciate the suggestion. We have thoroughly revised the manuscript and removed figure 1 from the study due to licensing issues. The removal was voluntary and the figure doesn't have much impact on the analysis.

12. How figure 2 is plotted?
Response: We appreciate this comment. Figure 2 represents the total population at risk of the three NTDs (LF, STH and SCH) across the eight regions in Kenya. We have also improved the clarity of the figure using the NAAS tool.

13. English throughout the manuscript need to be revisited.
Response: We appreciate this great observation. We have revisited the entire manuscript and polished the language.

14. Figures and the tables can be inclined along with the text. Why it is placed at the end of the paper?
Response: Thanks for this great observation. We have reformatted and moved all the figures and tables from the end of the document to the corresponding text within the document where they are first referenced.

15. Architecture about the proposed methodology may be drawn in the paper and placed in the methodology section.
Response: We appreciate this suggestion. We have added a flowchart (figure 1) in the methodology section showing the architecture of the model.
Lines 166 - 206

Reviewer 3:

Population Description

Population = Kenya subnational units with co-endemicity data (LF, SCH, STH). This is clearly defined and appropriate for testing the hypothesis. This could be improved by adding: total number of districts/units analyzed, and justification for inclusion/exclusion. How many geographic units were analyzed, and is this sufficient for model training/testing (especially with 80:20 split)?"

Response: We appreciate this great comment. We have added a section on population description (2.2) as shown below;

Lines 128 - 135

In this study, secondary data analysis using the Expanded Special Project for Elimination of Neglected Tropical Diseases (ESPEN) in Kenya's co - endemicty data for the year 2022 was done. Kenya, on the east coast of Africa, is endemic for LF, Schistosomiasis and STH. The Ministry of Health implements a Strategic Plan for control of NTDs through the National NTD control program. This study analyzed data at both national and subnational levels (implementation units) including a total of 47 counties and 290 implementation units which was sufficient for modelling.

Click here to access/download
Revised Article with Changes Highlighted
Revised Manuscript with Track Changes.docx
