# Supplementary material for: Advanced machine learning approaches for predicting Neglected Tropical Disease co-endemicity in Kenya: A focus on soil-transmitted helminths, schistosomiasis, and lymphatic filariasis
Source: PLoS Negl Trop Dis. 2026 Mar 30;20(3):e0014156. doi: 10.1371/journal.pntd.0014156 (PMC13046276; doi:10.1371/journal.pntd.0014156)
Supplement: Attachment — Submitted filename: Response to Reviewers.docx [file pntd.0014156.s005.docx]

**Advanced Machine Learning Approaches for Predicting Neglected Tropical Disease (NTD) Co-endemicity in Kenya: A Focus on Soil-Transmitted Helminthes, Schistosomiasis, and Lymphatic Filariasis.**

Reviewer Comments:

**Reviewer 1:**

1. Model comparison was done only using AUC? Why not with other parameters?

Response: We greatly appreciate the reviewer’s suggestion. We have added a table (table 9 ) summarizing all the key classification metrics for RF, GBM, and XGBoost on the test set including Accuracy, kappa, sensitivity, specificity and confidence intervals.

**Line 578-592 as shown below:**

**Table 9: Models performance based on selected metrics.**

| Metric | RF | GBM | XGBoost |
| --- | --- | --- | --- |
| ROC AUC | 0.7030 | 0.6200 | 0.6560 |
| Accuracy | 0.6842 | 0.6140 | 0.5965 |
| Sensitivity | 0.8387 | 0.7742 | 0.7419 |
| Specificity | 0.5000 | 0.4231 | 0.4231 |
| Kappa | 0.3473 | 0.2023 | 0.1687 |
| 95% CI | 0.5476,0.8009 | 0.4757,0.7400 | 0.4582,0.7244 |

With a value of 0.7030, RF obtained the highest Area under the Receiver Operating Characteristic Curve (ROC AUC), demonstrating the best ability to distinguish between the positive and negative classes. Significantly better than the performance of the other two models (Kappa of 0.2023 for GBM and 0.1687 for XGBoost), this superior discrimination translated into a noteworthy Accuracy of 0.6842 and a Kappa statistic of 0.3473, suggesting moderate agreement beyond chance. With ROC AUC values of 0.6200 and 0.6560, respectively, the GBM and XGBoost models performed similarly, falling short of the RF approach's predictive power.

2. The comparison graph AUC is not scaled properly and the graph can be much improved.

Response: Thank you for this great observation. We have fitted the graph to a proper scale and improved it accordingly. Additionally, we have used the PLOS’s NAAS tool to improve all the figures in the entire manuscript.

3. Why you have chosen only three ML algorithms namely RF, GBM and XG boost? Any specific reasons?

Response: Many thanks for this great concern. We have added a section in the methodology section (2.8) on “Algorithm Selection Justification” as shown below.

Lines 272 – 279.

The study chose only three machine learning algorithms namely RF, GBM and XGBoost. Firstly, the three algorithms are tree-based ensemble methods known for robustness to non-linear and complex relationships which are common in disease data, handling mixed data types i.e., categorical, continuous, discrete and without extensive feature scaling and lastly providing feature importance Scores (key for interpretability in disease modelling context). The Random forest and XGBoost are the leading tree-based ensembles selected for their well-documented performance on structured data and their ability to capture non-linearities.

4. Why other classification algorithms are not chosen? Give Reasons.

Response: We appreciate this question. We have addressed this in no.3 above whereby the algorithms were chosen purposefully due to their robustness to nonlinear and complex data.

5. What is the reason RF is giving good results compared to other algorithms take for experiment?

Response: Many thanks for this great concern. We have included a paragraph in the discussion section explaining the “surprising” results of the RF giving better results XGBoost and GBM.

Lines 611-621 as shown below.

Firstly, RF is less prone to over fitting than the other models because it uses bootstrap aggregating (bagging) and random subspace sampling. It is less sensitive to hyper parameter tuning and its design inherently handles noise/outliers better. GBM and XGBoost, on the other hand, employ boosting, training trees one after the other, fixing the mistakes (residuals) of the previous tree. They are more prone to over fitting and capturing noise when applied to unseen (test) data, even though this reduces bias and frequently produces excellent performance on the training set. In contrast to GBM and particularly XGBoost, which frequently need extensive and meticulous hyper parameter tuning to prevent over fitting and attain optimal generalization performance, RF is frequently very effective with its default hyper parameter settings. The boosting models' propensity to over fit the training data was probably not adequately controlled by the default or basic tuning that was used.

6. Whether the classes in the dataset are uniformly distributed?

Response: Thank you for this insightful input. We have included the class imbalance technique in the methodology section (section 2.4) as shown below.

Lines 158 – 164;

Data cleaning was carried out in R software version 4.5.0. Missing values for the disease data were detected during cleaning and were replaced using the mean of the complete samples. Since the data was balanced (35:65), there was no need of re-sampling the minority class. The data was balanced to a moderate extent to perform the training process to build the co - endemicity prediction.

7. How many features are in the dataset? Whether you considered all? No discussion in this regard.

Response: We appreciate this comment. We have added a sub section on feature selection and preparation, number of features selected, number of features removed and the criteria used in the methodology section (2.4) as shown below.

Lines 159-165;

Data cleaning was carried out in R software version 4.5.0. The dataset contained 37 variables which were exclusively selected using the variable importance criterion. The missing values for the diseases data were detected during cleaning and were replaced using the mean of the complete samples. Since the data was balanced (35:65), there was no need of re-sampling the minority class. The data was balanced to a moderate extent to perform the training process to build the co - endemicity prediction. Sections 2.5 and 2.6 give more details on the variables used in the study.

8. No details about the size of the dataset?

Response: Thank you for this great question. We have added the details about the size of the dataset including the data size, the number of geographic units, the number of features, and the final data split in the methodology section as shown below;

Lines 137-143

In this study, secondary data analysis using the Expanded Special Project for Elimination of Neglected Tropical Diseases (ESPEN) in Kenya’s co - endemicty data for the year 2022 was done. Kenya, on the east coast of Africa, is endemic for LF, Schistosomiasis and STH. The Ministry of Health implements a Strategic Plan for control of NTDs through the National NTD control program. This study analyzed data at both national and subnational levels (implementation units) including a total of 47 counties and 290 implementation units which was sufficient for modelling.

9. Why deep learning models are not preferred for the given problem?

Response: We appreciate this good question and concern. Deep Learning models (like CNNs or RNNs) were not used because they require a much larger dataset (millions of observations) to train effectively and lacks the interpretability (feature importance) crucial for this NTDs modeling task.

10. Details such as missing values, categorical variables and other details in exploratory data analysis are missing.

Response: We appreciate this addition. We have expanded the methodology section and added details on missing values, categorical variables and data cleaning.

Lines 159 - 165; 226 - 230.

11. Figure 1 need to be of high clarity. Currently it is not so. Please change. Highlight the area which is being focused in this research.

Response: We appreciate the suggestion. We have thoroughly revised the manuscript and removed figure 1 from the study due to licensing issues. The removal was voluntary and the figure doesn’t have much impact on the analysis.

12. How figure 2 is plotted?

Response: We appreciate this comment. Figure 2 represents the total population at risk of the three NTDs (LF, STH and SCH) across the eight regions in Kenya. We have also improved the clarity of the figure using the NAAS tool.

13. English throughout the manuscript need to be revisited.

Response: We appreciate this great observation. We have revisited the entire manuscript and polished the language.

14. Figures and the tables can be inclined along with the text. Why it is placed at the end of the paper?

Response: Thanks for this great observation. We have reformatted and moved all the figures and tables from the end of the document to the corresponding text within the document where they are first referenced.

15. Architecture about the proposed methodology may be drawn in the paper and placed in the methodology section.

Response: We appreciate this suggestion. We have added a flowchart (figure 1) in the methodology section showing the architecture of the model.

Lines 166 - 206

**Reviewer 3:**

Population Description

Population = Kenya subnational units with co-endemicity data (LF, SCH, STH). This is clearly defined and appropriate for testing the hypothesis. This could be improved by adding: total number of districts/units analyzed, and justification for inclusion/exclusion. How many geographic units were analyzed, and is this sufficient for model training/testing (especially with 80:20 split)?”

Response: We appreciate this great comment. We have added a section on population description (2.2) as shown below;

Lines 128 - 135

In this study, secondary data analysis using the Expanded Special Project for Elimination of Neglected Tropical Diseases (ESPEN) in Kenya’s co - endemicty data for the year 2022 was done. Kenya, on the east coast of Africa, is endemic for LF, Schistosomiasis and STH. The Ministry of Health implements a Strategic Plan for control of NTDs through the National NTD control program. This study analyzed data at both national and subnational levels (implementation units) including a total of 47 counties and 290 implementation units which was sufficient for modelling.
