# Supplementary material for: Advanced machine learning approaches for predicting Neglected Tropical Disease co-endemicity in Kenya: A focus on soil-transmitted helminths, schistosomiasis, and lymphatic filariasis
Source: PLoS Negl Trop Dis. 2026 Mar 30;20(3):e0014156. doi: 10.1371/journal.pntd.0014156 (PMC13046276; doi:10.1371/journal.pntd.0014156)
Supplement: Attachment — Submitted filename: Peer Review Report.docx [file pntd.0014156.s003.docx]

PEER REVIEW: #PNTD-D-25-01087R1

**Manuscript Title:** Advanced Machine Learning Approaches for Predicting Neglected Tropical Disease (NTD) Co-endemicity in Kenya: A Focus on Soil-Transmitted Helminths, Schistosomiasis, and Lymphatic Filariasis.

General Assessment

This manuscript addresses an important public health challenge in sub-Saharan Africa: the co-endemicity of neglected tropical diseases (NTDs). The integration of machine learning techniques with WASH and demographic data is timely and relevant, particularly for informing Kenya’s NTD Elimination Strategic Plan 2030. The study is generally well motivated and presents a substantial amount of empirical analysis. However, several methodological clarifications, structural refinements, and language improvements are necessary to strengthen scientific rigor, clarity, and reproducibility.

Minor Comments and Technical Issues

1. Language and Grammar: Numerous grammatical errors, spacing issues, content alignment (line 132-136) and typographical mistakes (e.g., “Machine learning offers”, inconsistent hyphenation of “co-endemicity”). Hence, Professional language editing is strongly advised.
2. Figures and Tables: Several figures are referenced but not clearly described or interpreted in the text. Some tables have mislabeled titles (e.g., Table 5 referring to RF instead of GBM).
3. Consistency: Ensure consistent use of terms (e.g., “XG Boost” vs “XGBoost”). Align section numbering (two Section 2.3s appear).
4. See the suggested keywords, remove the key word WASH, RF, and ESPEN.
5. Ethics Statement: Adequate, but could be shortened and moved to a standard “Ethics approval and consent” subsection per PLOS style.

**Major Concerns and Recommendations**

1. Although the Random Forest model outperforms others, overall AUC values (≈0.62–0.70) indicate only *moderate predictive ability*. Why?
2. Explicitly acknowledge limitations of moderate AUC values and potential misclassification risks.
3. Discuss implications of low specificity across models, especially for resource allocation.
4. The construction of the co-endemicity outcome variable combines multiple disease states into one categorical outcome, but the epidemiological rationale for grouping specific combinations is not sufficiently justified. Moreover, the number of observations per class is also not reported. With this regard, do the following;
i. Provide a table showing class frequencies.
ii. Justify the co-endemicity grouping scheme from a public health or intervention perspective.
5. Missing values were replaced using the mean of complete samples. This approach may bias estimates, especially for spatial and epidemiological data. I therefore, recommend the following two aspects;
i. Justify the choice of mean imputation.
ii. Discuss potential bias and consider sensitivity analysis or more robust imputation methods (e.g., k-NN or multiple imputation).
6. Clearly distinguish between tuned and final model configurations. Either, provide performance metrics for the ensemble model, or remove the ensemble section if it was not fully implemented.
7. Some correlation interpretations appear counterintuitive (e.g., positive correlation between improved water access and disease prevalence). These findings are not sufficiently explained. Therefore, provide epidemiological explanations (e.g., reporting bias, urban aggregation, ecological effects).
8. Reduce the repetitions of long forms of acronyms eg MDA
9. Clarify why correlation does not imply causation.

**Overall Recommendation: Minor Revision**

The manuscript has **strong potential** and addresses a critical gap in NTD co-endemicity modeling in Kenya. However, **minor** **revisions** are required to: Improve methodological transparency, Moderate claims based on model performance, Strengthen epidemiological interpretation, and Correct language and structural issues.

With these revisions, the manuscript would be a valuable contribution to the literature on data-driven NTD surveillance and control.
