# Supplementary material for: Advanced machine learning approaches for predicting Neglected Tropical Disease co-endemicity in Kenya: A focus on soil-transmitted helminths, schistosomiasis, and lymphatic filariasis
Source: PLoS Negl Trop Dis. 2026 Mar 30;20(3):e0014156. doi: 10.1371/journal.pntd.0014156 (PMC13046276; doi:10.1371/journal.pntd.0014156)
Supplement: Attachment — Submitted filename: Response_to_Reviewers_auresp_2.docx [file pntd.0014156.s006.docx]

**Advanced Machine Learning Approaches for Predicting Neglected Tropical Disease (NTD) Co-endemicity in Kenya: A Focus on Soil-Transmitted Helminthes, Schistosomiasis, and Lymphatic Filariasis.**

We sincerely thank the reviewers for the constructive comments and valuable suggestions that have helped improve the quality, clarity and scientific rigor of this manuscript. All comments have been carefully addressed and the manuscript has been revised accordingly.

A detailed point-by-point response is provided below.

MINOR COMMENTS

1. Language and grammar

Response: The manuscript has been carefully and thoroughly edited for grammar, clarity, flow and consistency. The specific corrections made in the manuscript include;

i. Correcting spacing errors (e.g., “Machine learningoffers” changed to “Machine learning offers”)
ii. Standardizing hyphenation of co-endemicity
iii. Correcting figure references and alignment issues e.g. figure 5
iv. Improving sentence structure throughout the introduction and discussion sections.
v. Section numbering corrected (duplicate Section 2.3 removed)

2. Figures and tables not clearly interpreted

Response: Additional explanations have been added in the Results section to better interpret figures and tables. Each figure is now clearly described in the text, highlighting its epidemiological relevance. In addition, Table 5 was corrected to ensure the title accurately reflects the GBM model results rather than RF, as originally mislabeled.

3. Suggested keywords

Response: The keywords have been revised as suggested. The terms WASH, RF, and ESPEN were removed to improve indexing relevance

4. Ethics statement placement

Response: The ethics statement has been shortened and moved to a separate subsection titled:

“Ethics approval and consent to participate”

in accordance with PLOS formatting guidelines.

MAJOR COMMENTS

1. Although the Random Forest model outperforms others, overall AUC values (≈0.62–0.70) indicate only *moderate predictive ability*. Why?

Response: We thank the reviewer for raising this important point. A paragraph has been added in the discussion section explaining the moderate predictive performance.

“Although the Random Forest model achieved the highest predictive performance (AUC = 0.703), the overall model performance can be considered moderate. Several factors may explain this outcome. First, the dataset consisted of a limited number of implementation units (n = 290) and a relatively small number of predictors, which may constrain the model’s ability to capture complex transmission dynamics. Second, NTD transmission is influenced by multiple environmental, climatic, socioeconomic, and behavioral factors that were not fully captured in the available dataset. Third, the co-endemicity outcome variable combines multiple disease states into a single classification framework, which increases classification complexity and may reduce predictive discrimination.”

2. Explicitly acknowledge limitations of moderate AUC values and potential misclassification risks.

Response: We sincerely appreciate this insightful comment. A new paragraph has been added to the Study Limitations section.

“Another limitation relates to the moderate predictive performance of the models (AUC range: 0.62–0.70). While these values indicate predictive ability above random classification, they also suggest that some misclassification may occur when identifying high-risk areas. Therefore, model outputs should be interpreted as decision-support tools rather than definitive predictors of disease transmission”.

3. Discuss implications of low specificity across models, especially for resource allocation.

Response: This comment is greatly appreciated. As such, a new explanation has been added in the discussion section.

“The relatively low specificity observed across the models indicates that some low-risk areas may be incorrectly classified as high-risk. From a public health perspective, this may lead to over-allocation of resources to certain areas. However, in disease control programs, prioritizing sensitivity (detecting true high-risk areas) is often preferred because failing to identify high-transmission regions could undermine elimination efforts”

4. The construction of the co-endemicity outcome variable combines multiple disease states into one categorical outcome, but the epidemiological rationale for grouping specific combinations is not sufficiently justified. Moreover, the number of observations per class is also not reported. With this regard, do the following;
i. Provide a table showing class frequencies.
ii. Justify the co-endemicity grouping scheme from a public health or intervention perspective.

Response: This great idea is highly appreciated. The rationale for grouping disease combinations has been clarified.

The grouping of diseases into co-endemicity categories was guided by public health intervention strategies used in preventive chemotherapy programs. Many NTD control programs implement integrated mass drug administration where treatment packages target multiple infections simultaneously. Therefore, modeling combinations of LF, SCH, and STH provides a more realistic representation of intervention planning and resource allocation.

Table 2: Frequency distribution of co-endemicity classes

| **Co-endemicity class** | **Frequency** | **Percentage** |
| --- | --- | --- |
| LF + STH + SCH | 2 | 0.69% |
| LF + STH | 3 | 1.03% |
| LF + SCH | 3 | 1.03% |
| STH + SCH | 16 | 5.52% |
| Single disease | 119 | 41.03% |
| Non endemic | 147 | 50.69% |

**The non-endemic areas dominate (50.69%)**, meaning more than half of the implementation units have none of the three diseases. **Single disease endemicity (41.03%)** is also common, indicating that many areas face only one of the diseases. **Dual co-endemicity** is relatively low (**STH + SCH** is the most frequent combination (5.52%)). **Triple co-endemicity (LF + STH + SCH)** is **very rare (0.69%)**, suggesting limited overlap of all three diseases.

5. Missing values were replaced using the mean of complete samples. This approach may bias estimates, especially for spatial and epidemiological data. I therefore, recommend the following two aspects;
i. Justify the choice of mean imputation.
ii. Discuss potential bias and consider sensitivity analysis or more robust imputation methods (e.g., k-NN or multiple imputation).

Response: This great input is highly appreciated. We have added justification in the methodology and limitation sections.

In the methodology section, “Missing values were replaced using mean imputation because the proportion of missing observations was relatively small and this approach allows preservation of the dataset size without introducing additional model complexity. Mean imputation is commonly used in exploratory machine learning analyses where missingness is minimal” has been added.

“Mean imputation may introduce bias by underestimating variability in the dataset. Future work could explore more robust imputation approaches such as k-nearest neighbors (k-NN) or multiple imputation to improve data representation” was added in the limitations section.

6. Clearly distinguish between tuned and final model configurations. Either, provide performance metrics for the ensemble model, or remove the ensemble section if it was not fully implemented.

Response: Many thanks for this great concern. The manuscript has been revised to clearly distinguish between Hyperparameter tuning stage and the final model configuration. Additionally, the ensemble modeling section was clarified.

“Hyperparameter tuning was performed using cross-validation to identify optimal model parameters. The final models were then trained using the selected parameters and evaluated on the independent test dataset”.

We have also removed the ensemble section.

7. Some correlation interpretations appear counterintuitive (e.g., positive correlation between improved water access and disease prevalence). These findings are not sufficiently explained. Therefore, provide epidemiological explanations (e.g., reporting bias, urban aggregation, ecological effects).

Response: Many thanks for this input. An explanation has been added as described in the paragraph below;

“The positive correlation observed between improved water access and disease prevalence may appear counterintuitive. However, this relationship may reflect ecological and reporting biases. For example, regions with improved infrastructure often have stronger surveillance systems, resulting in higher detection rates of infections. Additionally, urban aggregation effects and population density may increase exposure opportunities despite improved water access”.

8. Reduce the repetitions of long forms of acronyms eg MDA

Response: Thanks for this great observation. Long forms such as Mass Drug Administration (MDA) are now written once and abbreviated thereafter.

9. Clarify why correlation does not imply causation.

Response: Thanks for this important question. We have added clarification in the statistical analysis section.

“It is important to note that correlation analysis identifies statistical associations between variables but does not imply causal relationships. Therefore, the observed correlations should be interpreted cautiously and in conjunction with epidemiological evidence”.
